# Combining spectral and texture feature of UAV image with plant height to improve LAI estimation of winter wheat at jointing stage

**DOI:** 10.3389/fpls.2023.1272049

**Published:** 2024-01-03

**Authors:** Mengxi Zou, Yu Liu, Maodong Fu, Cunjun Li, Zixiang Zhou, Haoran Meng, Enguang Xing, Yanmin Ren

**Affiliations:** ^1^ College of Geomatics, Xi’an University of Science and Technology, Xi’an, China; ^2^ Research Center of Information Technology, Beijing Academy of Agriculture and Forestry Science, Beijing, China; ^3^ Hebei Maodong Xingteng Agricultural Technology Service Co., Ltd, Cangzhou, China; ^4^ Qingyuan Smart Agriculture and Rural Research Institute, Qingyuan, China

**Keywords:** plant height, feature fusion, machine learning, deep learning, UAV, LAI, winter wheat

## Abstract

**Introduction:**

Leaf area index (LAI) is a critical physiological and biochemical parameter that profoundly affects vegetation growth. Accurately estimating the LAI for winter wheat during jointing stage is particularly important for monitoring wheat growth status and optimizing variable fertilization decisions. Recently, unmanned aerial vehicle (UAV) data and machine/depth learning methods are widely used in crop growth parameter estimation. In traditional methods, vegetation indices (VI) and texture are usually to estimate LAI. Plant Height (PH) unlike them, contains information about the vertical structure of plants, which should be consider.

**Methods:**

Taking Xixingdian Township, Cangzhou City, Hebei Province, China as the research area in this paper, and four machine learning algorithms, namely, support vector machine(SVM), back propagation neural network (BPNN), random forest (RF), extreme gradient boosting (XGBoost), and two deep learning algorithms, namely, convolutional neural network (CNN) and long short-term memory neural network (LSTM), were applied to estimate LAI of winter wheat at jointing stage by integrating the spectral and texture features as well as the plant height information from UAV multispectral images. Initially, Digital Surface Model (DSM) and Digital Orthophoto Map (DOM) were generated. Subsequently, the PH, VI and texture features were extracted, and the texture indices (TI) was further constructed. The measured LAI on the ground were collected for the same period and calculated its Pearson correlation coefficient with PH, VI and TI to pick the feature variables with high correlation. The VI, TI, PH and fusion were considered as the independent features, and the sample set partitioning based on joint x-y distance (SPXY) method was used to divide the calibration set and validation set of samples.

**Results:**

The ability of different inputs and algorithms to estimate winter wheat LAI were evaluated. The results showed that (1) The addition of PH as a feature variable significantly improved the accuracy of the LAI estimation, indicating that wheat plant height played a vital role as a supplementary parameter for LAI inversion modeling based on traditional indices; (2) The combination of texture features, including normalized difference texture indices (NDTI), difference texture indices (DTI), and ratio texture indices (RTI), substantially improved the correlation between texture features and LAI; Furthermore, multi-feature combinations of VI, TI, and PH exhibited superior capability in estimating LAI for winter wheat; (3) Six regression algorithms have achieved high accuracy in estimating LAI, among which the XGBoost algorithm estimated winter wheat LAI with the highest overall accuracy and best results, achieving the highest R^2^ (R^2 = ^0.88), the lowest RMSE (RMSE=0.69), and an RPD greater than 2 (RPD=2.54).

**Discussion:**

This study provided compelling evidence that utilizing XGBoost and integrating spectral, texture, and plant height information extracted from UAV data can accurately monitor LAI during the jointing stage of winter wheat. The research results will provide a new perspective for accurate monitoring of crop parameters through remote sensing.

## 1 Introduction

Winter wheat is the second-largest grain crop in China in terms of cultivated area and total output (Han, 2011), and it holds significant economic value. Investigating the agronomic parameters of winter wheat is essential to agricultural production management, especially to enhance grain production. Leaf Area Index (LAI) stands as a crucial agronomic parameter for winter wheat, which is defined as the ratio of total plant leaf area per unit of land area to land area. LAI is directly related to crop growth (Casa et al., 2012) and serves as a vital indicator for monitoring crop growth, biomass estimation, and pre-harvest yield prediction during the fertility period (Pinter et al., 2003; Dente et al., 2008). In the context of production management, wheat topdressing during the jointing stage is pivotal to improving yield and quality. China has implemented a zero-growth policy for chemical fertilizers and pesticides (Cui et al., 2021), alongside developing variable rate fertilizer applicators to enable precise fertilizer application based on local conditions. Therefore, the accurate and rapid estimation of LAI for winter wheat at jointing stage is not only conducive to the real-time monitoring of crop growth and development, but also has important significance for the formulation of variable rate fertilization prescription for agricultural machinery, reducing the use of chemical fertilizer and mitigating soil pollution.

The measurement methods of LAI include direct and indirect approaches. The direct method is a traditional and destructive method, mainly through manual field observation, which is time-consuming and laborious (Hu et al., 2018). The indirect method employs optical instruments or remote sensing inversion, offering a convenient and fast approach (Torres-S´anchez et al., 2014). Among these methods, remote sensing technology has gained widespread adoption as an indirect means of monitoring agronomic parameters (Zhang et al., 2008). Remote sensing platforms can be categorized into ground, aerial and space remote sensing platform based on their height above the ground (Lu et al., 2022). Ground remote sensing platforms mainly employ spectrograph for measurement. However, due to the height restrictions, these platforms face the challenge of obtaining Digital Orthophoto Maps (DOM) and monitor large-scale areas efficiently (Behrens and Diepenbrock, 2006; Nie et al., 2016). Space remote sensing platforms mainly rely on satellites to acquire data, enabling the monitoring large areas. Nevertheless, factors such as satellite revisit time and atmospheric conditions often hinder meeting the demands for spatiotemporal resolution (Liu et al., 2012). An alternative approach is the aerial operation mode, which employs unmanned aerial vehicle (UAV) (Li et al., 2019) for remote sensing. Compared to manned aircraft, UAV remote sensing has the advantages of low cost, simple operation and strong flexibility (Hassan et al., 2019). Furthermore, the multispectral sensors carried by UAV provide more bands than digital camera sensors, and the spectral information avoids data redundancy seen in hyperspectral sensors, which can be effectively applied to monitor crop LAI (Sun et al., 2019).

Currently, there are two implementations for monitoring crop LAI using UAV multispectral remote sensing. One is a radiative transfer physical model, and the other is a statistical empirical model. The physical model is based on the reflection and absorption between light and crops, which has a certain mechanism and strong versatility (Fu et al., 2022a). However, the model involves complex formulas and requires many parameters, which makes it difficult to find the optimal solution (Du et al., 2016). In contrast, the empirical model establishes the relationship between UAV image features and winter wheat growth parameters through statistical methods (Liang et al., 2015; Cao et al., 2020). This method proves to be straightforward and user-friendly, estimating LAI by analyzing the statistical relationship between raw spectra or extracted vegetation indices and ground-based measured LAI data. Nonetheless, apart from spectral features, UAV multispectral images also provide abundant texture information associated with vegetation growth (Kupidura, 2019). Texture information reflect inherent characteristics of the image, making it essential to consider them when extracting vegetation growth parameters from multispectral images. Some scholars have successfully estimated LAI of rice (Cao et al., 2022), potato (Li et al., 2023), sorghum (Potgieter et al., 2017) and other crops, as well as biomass (Dai et al., 2022) and yield (Fu et al., 2020) of winter wheat by integrating spectral and texture features, providing promising results. However, previous studies have mostly directly input a large number of texture features into the model for training, lacking the optimization of texture features. To address this limitation, a more refined and selective approach should be considered to enhance the effectiveness and accuracy of integrating texture information into the model (Zhu et al., 2019). Texture features provide valuable information about the small-scale structures and details in an image. The optimization of extraction methods plays a pivotal role in achieving a more precise capture of structural information within the image, consequently elevating the accuracy of image analysis. Simultaneously, this optimization process contributes to the enhancement of the level of detail present within the image, rendering it richer in information content and enhancing the effectiveness and accuracy of integrating texture information into the model. For instance, the optimized texture indices can simultaneously capture the influence of two distinct texture features on wheat LAI monitoring (Zhang et al., 2022b).

The spectral and texture information extracted from the UAV multispectral image only contains the crop canopy information. During the period of crop growth, the vegetation indices are not sensitive to the changes in canopy information, and the spectral signal may become saturated (Liu et al., 2018), thereby affecting the accuracy of LAI estimation to some extent. Recent studies have revealed a significant correlation between crop canopy height and LAI (Liu et al., 2022). Notably, extracting plant height from UAV based on canopy height model (CHM) can alleviate the issue of spectral saturation. Niu et al. (Niu et al., 2018) have demonstrated improvements in LAI estimation accuracy by fusing plant height data with UAV digital image variables for maize breeding materials, surpassing the performance of using only digital image variables. Gao et al. (Gao et al., 2020) have successfully enhanced the inversion accuracy of LAI by combining crop height parameters with vegetation indices. Therefore, it is necessary to incorporate crop vertical structure characteristics, such as height, into the inversion process of winter wheat LAI to get more accurate and reliable results.

Generally speaking, researchers commonly use empirical or semi empirical models, employing statistical regression analysis of spectral features, to construct LAI estimation models. With the development of crop LAI inversion research, some researchers have begun exploring the utilization of machine learning (ML) techniques to build LAI inversion model and enhance estimation accuracy. Partial Least Square Regression (PLSR) (Hasan et al., 2019), Support Vector Machine (SVM) (Azadbakht et al., 2019), Random Forest (RF) (Zhang et al., 2018), Extreme Gradient Boosting (XGBoost) (Zhang et al., 2021c), and other machine learning algorithms are commonly used for wheat LAI inversion. Furthermore, deep learning (DL), a sophisticated machine learning algorithm, has gained traction in crop yield estimation and prediction, with convolutional neural network (CNN) and recursive neural network (RNN) being widely applied in related studies (Khaki et al., 2019; Koirala et al., 2019). Long short-term memory (LSTM) neural network is an improved RNN with a special recursive structure and gating mechanism, which can adjust the information in and out of the unit, and has high prediction accuracy in the field of wheat yield and biomass estimation (Wang et al., 2022a). These ML-based methods operate on diverse model frameworks, through learning from the training set data to construct the inversion model, thereby establishing the relationship between predictor variables and response variables. Leveraging robust data analysis capabilities and achieving high estimation accuracy, ML approaches effectively circumvent the shortcomings of empirical or semi-empirical models prone to pathological issues (Pearson and Miller, 1972).

The above studies have achieved high estimation accuracy using various methods, which the R^2^ of the optimal models was range from 0.74 to 0.78. However, there remains a lack of research in exploring the potential of using ML/DL to improve the accuracy of winter wheat LAI estimation by combining spectral features, optimized texture features, and plant height based on UAV multispectral image. In light of this, the main objectives of the study were (1) to examine the influence of plant height on winter wheat LAI estimation during the jointing stage; (2) to evaluate the disparities in multi-feature estimation of winter wheat LAI with combinations of vegetation indices (VI), texture indices (TI), and plant height (PH); and (3) to conduct a comparative analysis of performances in the inversion modeling of winter wheat LAI with six regression algorithms, including support vector machine (SVM), back propagation neural network (BPNN), random forest (RF), extreme gradient boosting (XGBoost), convolutional neural network (CNN) and long short-term memory neural network (LSTM). The achievement of these objectives will provide a more reliable foundation for winter wheat LAI estimation, offering more precise monitoring and decision support for agricultural production.

## 2 Materials and methods

### 2.1 Study area and experimental design

**Figure 1:**
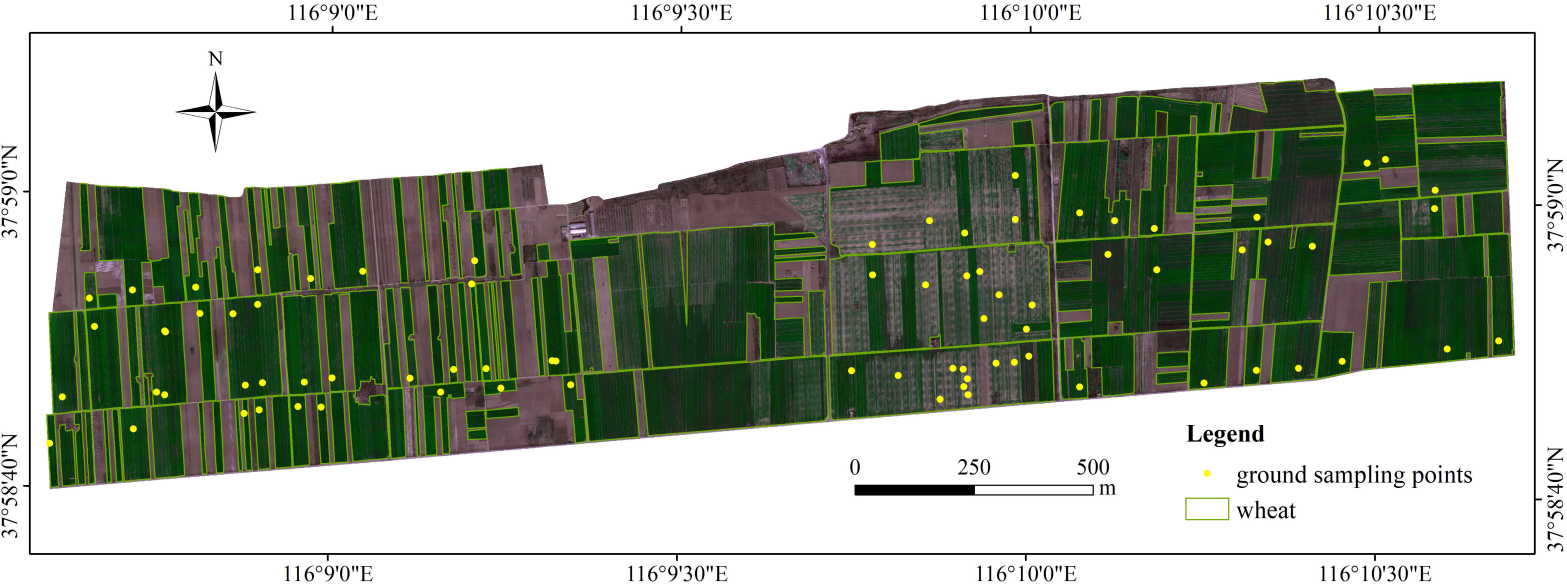
Study area of winter wheat LAI estimation experiment using UAV images.

Geographic Location: The study area is located in Xixindian Township, Cangzhou City, Hebei Province, China. The experimental field spans a geographic range from 116°08'38" E to 116°10'42" E longitude, and 37°58'40" N to 37°59'09" N latitude (**Figure 1**).

Climate: This region exhibits a warm temperate continental monsoon climate, characterized by pleasant temperatures. The annual average temperature is 12.7°C. Abundant sunlight graces the area, with an average annual sunshine duration of approximately 2.78 h. Precipitation predominantly occurs during the summer months, totaling around 543 mm of rainfall per year. The favorable climatic conditions in the region provide conducive circumstances for the growth and development of winter wheat. The above statistics are based on Government of the People&’s Republic of China information published in 2022.

Topography: The topography is flat, providing an ideal environment for the cultivation of diverse crops. The topographical conditions may influence the distribution and drainage of water, thereby impacting the growth conditions of winter wheat. Different topographical features may result in variations in soil moisture across different regions, consequently affecting the estimation of LAI.

Cropping System: Notably, summer maize and winter wheat serve as the primary cash crops within the experimental area, employing a rotational planting system where winter wheat is planted after the maize harvest.

Varieties and Practices: Due to the lack of standardized management practices among winter wheat farmers in the region, there is a significant variation in the selection of winter wheat varieties, irrigation levels, and fertilizer application. Different characteristics among varieties may lead to variations in LAI. Because distinct winter wheat varieties may exhibit differences in growth rates, leaf quantities, and structures, thereby influencing the estimation of their LAI. In this paper, the prominent winter wheat varieties cultivated include Jimai 22, Jimai 518, Luyuan 502, Shandong 20, and Tumai 6, each with distinct seeding rates ranging from 225 kg/ha to 300 kg/ha. The predominant irrigation methods employed are surface irrigation and sprinkler irrigation. In addition, different types and ratios of fertilizers may significantly impact leaf growth, leaf area, and photosynthesis. For example, fertilizers with high nitrogen content may stimulate leaf growth, consequently affecting the estimated values of LAI. The primary fertilizers utilized are Tuboshi (N-P_2_O_5_-K_2_O, 28-6-6) with ≥40% total nutrients, Jindadi (N-P_2_O_5_-K_2_O, 19-15-6) with ≥40% total nutrients, and Xishouliang (N-P_2_O_5_-K_2_O, 17-23-5) with ≥45% total nutrients. Across all varieties, the standard fertilizer application rate during cultivation is 600 kg/ha.

### 2.2 Data collection and preprocessing

#### 2.2.1 UAV image acquisition and pre-processing

In this experiment, due to its compatibility with the research objectives, the eBee SQ precision agriculture UAV equipped with the Parrot Sequoia multispectral sensor was selected. The Parrot Sequoia sensor was capable of capturing spectral data in four bands simultaneously, including green (550nm), red (660nm), red edge (735nm), and near-infrared (790nm) (Handique et al., 2017). Prior to the experiment, it was crucial for the success of the study to conduct flight planning using specialized software to determine the flight routes and parameters, including a flight altitude of 95.5m, a ground resolution of 9cm and an 80% overlap in both along-track and across-track directions. The UAV was launched using a hand-throw method, and before take off, take photos of the radiation correction plate and ensure the absence of shadows on the calibration board. The data acquisition was conducted on October 21, 2020 (at bare soil stage) and March 31, 2021 (at jointing stage) between 10:00 and 14:00 to reduce the influence of the changes in the solar altitude angle on the experiments. In order to minimize optical distortions and ensure clear image acquisition, the weather conditions were clear skies, without any clouds, and a gentle breeze throughout the entire data collection period.

The acquired UAV multispectral remote sensing data were preprocessed, which primarily included image correction, image mosaic, image clipping and image resampling. Pix4DMapper and ArcGIS 10.4 are widely-used software tools in the fields of Remote Sensing (RS) and UAV data processing. In this study, the UAV multispectral images were geometrically corrected and stitched with Pix4DMapper software to generate Digital Surface Model (DSM) and Digital Orthophoto Map (DOM), so as to obtain the spectral reflectance data and height data of the study area. After clipping based on the vector boundaries of the study area in ArcGIS 10.4 software, the image resolution was resampled to 0.1m using the cubic convolution difference method, aiming to ensure a sufficiently high detail resolution to accurately capture vegetation features.

#### 2.2.2 Ground data collection

The ground data collection was synchronized with the acquisition of UAV multispectral remote sensing data, and mainly included the value of PH and LAI, coordinate data of sample points and control points. The measurements of PH and LAI were primarily conducted for the establishment and validation of estimation models. The sample point coordinates involved recording the positions where PH and LAI were measured. On the other hand, control points were established to provide ground truth coordinates, facilitating the correlation with UAV data. Specifically, data were collected from 79 sampling points evenly distributed in the study area to ensure comprehensive coverage. This uniform distribution helped capture spatial variations in vegetation, enhancing the representativeness and reliability of the data.

Wheat is conventionally sown at approximately 15 cm intervals. This seeding technique plays a pivotal role in ensuring effective soil surface coverage, mitigating soil moisture evaporation, and consequently, fostering water conservation and yield augmentation. Therefore, during the measurement of PH(unit: cm), three representative wheat plants that encapsulate the comprehensive growth status at the sampling point are meticulously selected within a 50 cm radius around the designated point. A tape measure was used to measure the vertical height of each wheat plant, and the average value was taken as the PH of each sample point to ensure the data accuracy.

The measurement of LAI was conducted using the LAI-2200C plant canopy analyzer. The steps were taken to ensure data accuracy as follows. Prior to measurement, the instrument was aligned with the sun to determine the incident light intensity. During measurement, efforts were made to keep the instrument as horizontally aligned as possible. Three measurements were taken at each sampling point, and the average value was considered as the final LAI. LAI in the sample dataset ranged from 0.61 to 8.57, the average was 3.89 and the standard deviation was 1.93. Simultaneously, the HI-TARGET iRTK2 was employed for Real-Time Kinematic (RTK) measurements, allowing the acquisition of coordinates for each sample point and control point.

#### 2.2.3 Winter wheat pixels extraction

The green, red, near-infrared and red edge bands of UAV multispectral images, as well as normalized difference vegetation index (NDVI), enhanced vegetable index 2 (EVI2), red edge optimized soil-adjusted vegetation index (REOSAVI) and optimized soil-adjusted vegetation index (OSAVI) were used to input into the random forest classifier to extract winter wheat pixels (Fu et al., 2022b). NDVI is widely applied to reflect vegetation growth conditions. EVI2 considers atmospheric correction and soil influences, providing more accurate monitoring in areas with high vegetation cover. REOSAVI is an index optimized for the red-edge band that accounts for soil influences. OSAVI is a soil-adjusted vegetation index optimized to reduce the impact of the soil surface. Its reduction of soil effects in high vegetation density environments contributes to a more accurate assessment of vegetation conditions. Random Forest classifier is capable of efficiently handling large-scale datasets, exhibiting high classification accuracy, and is particularly well-suited for pixel-level image classification tasks. This study comprehensively assessed the quality of pixel extraction using overall accuracy and the kappa coefficient. Overall accuracy measures the overall precision of the classification results, while the kappa coefficient provides sensitivity to random errors and omissions in the classification. These metrics reflect the accuracy of the extraction process. The overall accuracy and kappa coefficient were 98.74% and 91.21%, respectively. ENVI 5.3 is software designed for remote sensing data analysis and image processing. Then this process was implemented in ENVI 5.3, with all parameters set to their default values.

### 2.3 LAI Estimation input features

#### 2.3.1 Vegetation indices

Vegetation indices (VI) are established by using the relationship between spectral data and various physical and chemical parameters of vegetation, which can effectively reflect the growth status of plants, and are widely used in the monitoring of physiological and biochemical parameters of plants. Generally, it is obtained by selecting two or more band reflectance data from spectral data and performing a series of combined operations such as addition, subtraction, multiplication and division. Compared with a single band, the band combination method is not only more sensitive to vegetation characteristics, but also can eliminate environmental background noise to a certain extent (Huete et al., 1985). At present, there are many kinds of vegetation indices, such as the difference vegetation index (DVI) and enhanced vegetable index2 (EVI2), which can control the impact of soil and environmental background. Simultaneously, red edge renormalized difference vegetation Index (RERDVI) primarily focuses on the structure and coverage of vegetation, aiding in understanding the spatial distribution and density of vegetation. Red edge chlorophyll Index (Clre) reflects variations in chlorophyll content within vegetation, serving to assess the growth status and overall health of the vegetation. Based on the previous research results, this paper selected 17 vegetation indices with great effect for retrieving wheat LAI, which were divided into greenness indices, structure indices and pigments indices, according to their main functions, as shown in **Table 1**. The size of the range of spectra measured at each sampling point was 10×10cm.

**Table 1:** VI used in this study.

Vegetation Index		Calculations	References
Greenness Indices	Ratio Vegetation Index	$RVI=\frac{NIR}{R}$	Pearson and Miller, 1972
	Difference Vegetation Index	DVI=NIR−R	Becker and Choudhury, 1988
	Normalized Difference Vegetation Index	$NDVI=\frac{NIR-R}{NIR+R}$	Rouse et al., 1974
	Green Normalized Difference Vegetation Index	$GNDVI=\frac{NIR-G}{NIR+G}$	Gitelson et al., 1996
	Enhanced Vegetation Index2	$EVI2=\frac{2.5\times \left(NIR-R\right)}{NIR+2.4\times R+1}$	Jiang et al., 2008
	Green Chlorophyll index	$CLgreen=\frac{NIR}{G-1}$	Gitelson et al., 2006
Structure Indices	Modified simple ratio	$MSR=\frac{\frac{NIR}{R}-1}{\sqrt{\frac{NIR}{R}}+1}$	Chen and Cihlar, 1996
	Modified Soil Adjusted Vegetation Index	$MSAVI=0.5\times \left[\begin{array}{l} \left(2\times NIR+1\right)-\\ {\left(\begin{array}{l} {\left(2\times NIR+1\right)}^{2}\\ -8\times \left(NIR-R\right) \end{array}\right)}^{0.5} \end{array}\right]$	Daughtry et al., 2000
	Green Optimized soil-adjusted vegetation index	$GOSAVI=1.16\times \frac{NIR-G}{NIR+G+0.16}$	Gilabert et al., 2002
	Red Edge Optimized soil-adjusted vegetation index	$REOSAVI=1.16\times \frac{NIR-R}{NIR+R+0.16}$	Kim et al., 1994
	Red Edge Renormalized difference vegetation Index	$RERDVI=\frac{NIR-RE}{NIR+RE}$	Kim et al., 1994
Chlorophyll Content Indices	Red edge Chlorophyll Index	$CLre=\frac{NIR}{RE-1}$	Gitelson et al., 2006
	Chlorophyll Absorption Ratio Index	CARI=(RE−R)−0.2×(RE+R)	Rondeaux et al., 1996
	Normalized Green Red Difference Index	$NGRDI=\frac{G-R}{G+R}$	Potgieter et al., 2017
	Triangular Vegetation Index	TVI=60×(NIR−G)−100×(R−G)	Broge and Leblanc, 2001
	Modified triangular vegetation index 2	$MTVI2=\frac{1.5\times \left(\begin{array}{l} 1.2\times \left(NIR-G\right)\\ -2.5\times \left(R-G\right) \end{array}\right)}{\sqrt{\begin{array}{l} {\left(2\times NIR+1\right)}^{2}-\\ \left(6\times NIR-5\times \sqrt{R}\right)-0.5 \end{array}}}$	Haboudane, 2004
	MERIS Terrestrial Chlorophyll Index	$MTCI=\frac{NIR-RE}{RE-R}$	Dash and Curran, 2004

#### 2.3.2 Texture indices

Richer texture information related to plant growth can be extracted from UAV multispectral images. Texture features are distinct from image attributes such as grayscale and color, it is represented by the grayscale distribution of pixels and their surrounding spatial neighbors. It is the reflection of the internal characteristics of plant on remote sensing images. It helps to reveal the details of vegetation structure, including the arrangement and density of leaves, which directly affect the estimation of LAI. At present, the most widely used is the gray level co-occurrence matrix (GLCM) of statistical analysis method, which has rotation invariance, multi-scale characteristics and low computational complexity. In this study, GLCM was utilized to extract texture features from the green, red, red edge, and near-infrared bands of the multispectral imagery in ENVI 5.3 software. Specifically, eight texture features were extracted from each band, including mean (MEA), variance (VAR), homogeneity (HOM), contrast (CON), dissimilarity (DIS), entropy (ENT), second moment (SEC), and correlation (COR), resulting in a total of 32 texture features. MEA provides insights into the overall trends of vegetation distribution and brightness, while VAR reveals the degree of dispersion between pixel grayscale levels in GLCM. HOM measures the uniformity of vegetation texture, CON captures brightness variation, DIS assesses dissimilarity in texture, ENT indicates texture complexity, SEC reflects overall texture trends, and COR measures the linear relationship between vegetation structures. The selection of these texture features is based on their sensitivity to vegetation structure and their proven performance in previous studies on texture analysis. In the course of extraction, the inter-pixel offset was established at a distance of one pixel, with preference given to the utilization of 3×3 window. Grayscale gradation was set to 64, while the angle parameter retained its default value.

Similar to the calculation of vegetation indices, based on the 32 texture features obtained above, the texture indices can be calculated by combining two different features (Zheng et al., 2018). The three kinds of texture indices used in this paper are normalized difference texture indices (NDTI), difference texture indices (DTI) and ratio texture indices (RTI). Matlab r2021a is a kind of software widely used in data processing. It was used to calculate them and 1984 TI were produced.


(1) $$NDTI=\frac{T_{1}-T_{2}}{T_{1}+T_{2}}$$

(2) $$DTI=T_{1}-T_{2}$$

(3) $$RTI=\frac{T_{1}}{T_{2}}$$

where *T_1_* and *T_2_* represent the random texture features of any band in the green, red, red edge and near-infrared bands respectively.

#### 2.3.3 Plant height

**Figure 2:**
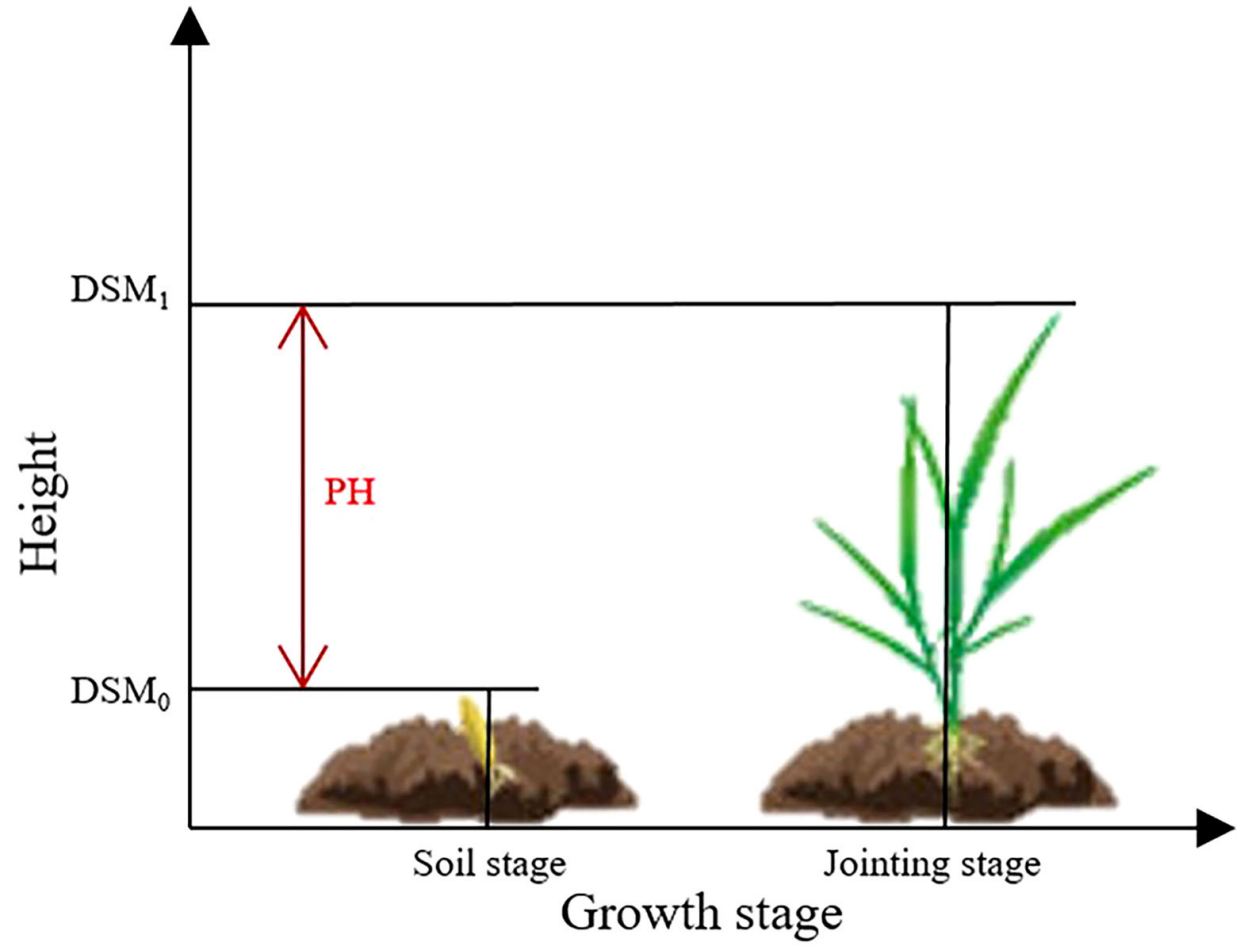
Principle of height extraction based on DSM.

The PH of winter wheat was extracted by generating DSM using UAV multispectral images during the bare soil stage and jointing stage in the study area. The DSM generated during the bare soil stage was labeled as DSM_0_, while the DSM generated during the jointing stage was labeled as DSM_1_. The PH was obtained by calculating the difference between DSM_1_ and DSM_0_, with DSM_0_ serving as the reference baseline (**Figure 2**).


(4) $$PH=DSM_{1}-DSM_{0}$$

### 2.4 LAI estimation with UAV images and accuracy verification

**Figure 3:**
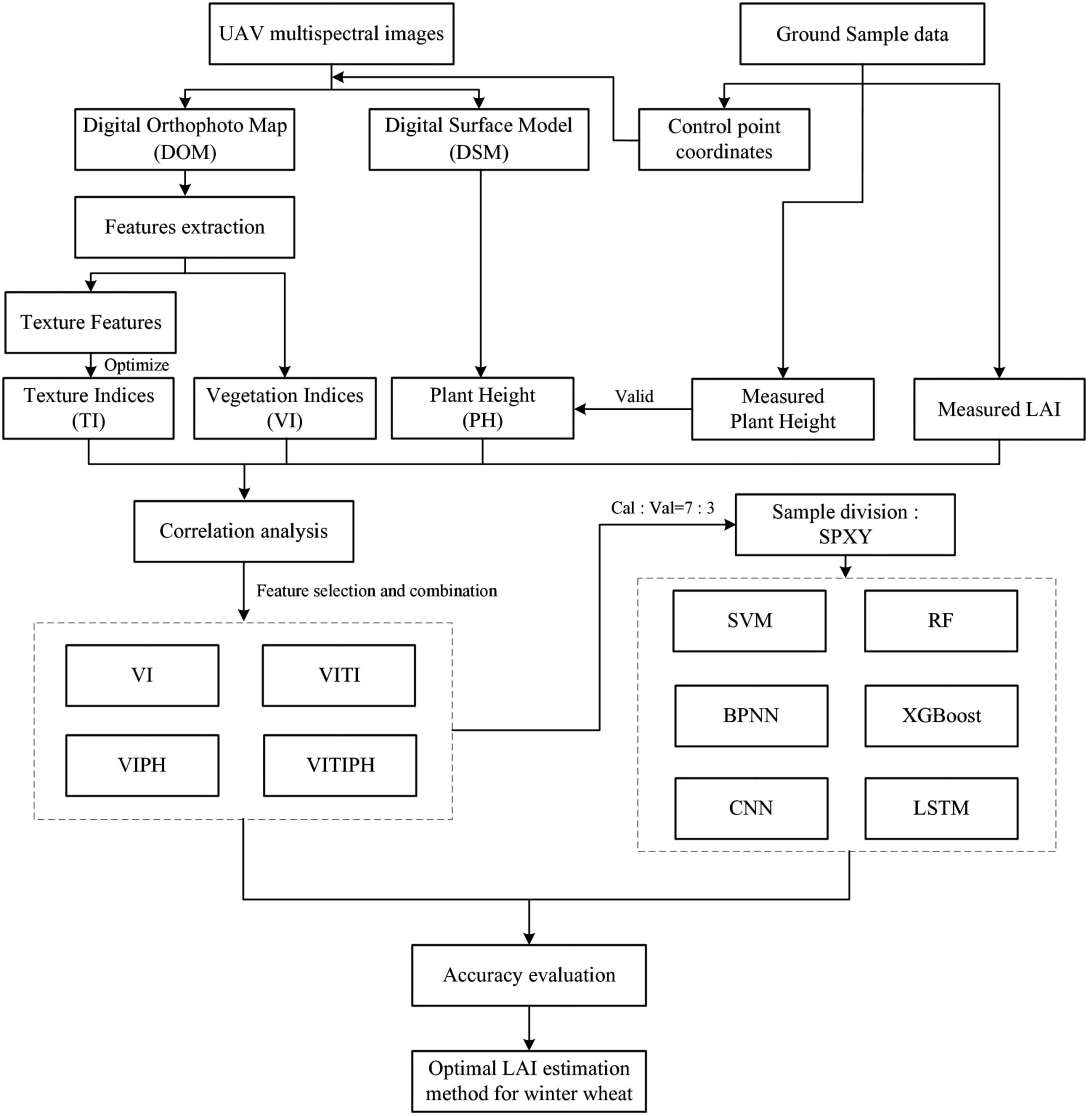
The technical route of winter wheat LAI estimation at jointing stage using UAV multispectral images.

The technical route of winter wheat LAI estimation at jointing stage using UAV multispectral images is shown in **Figure 3**.

#### 2.4.1 LAI Estimation algorithms

The correlation between the above indices and LAI was analyzed. Vegetation indices and texture indices with strong correlations (VI, TI), along with PH, were chosen as independent variables. The measured LAI on the ground was considered as the dependent variable. Different machine learning algorithms (RF, XGBoost, SVM, BPNN) and two deep learning algorithms (CNN and LSTM) were employed to explore the potential of combining spectral and texture features with PH for the winter wheat LAI estimation. The selection of these algorithms is based on their widespread application and success in handling complex nonlinear relationships, feature extraction, and generalization performance. SVM and RF were chosen for their excellence in capturing nonlinear patterns, while BPNN was favored for its ability to model intricate relationships. XGBoost was considered a robust regression algorithm due to its outstanding performance in handling high-dimensional data, mitigating overfitting, and improving overall model performance. As for deep learning algorithms, CNN and LSTM were selected to extract spatial and temporal features from multispectral images captured by UAV, providing a more comprehensive understanding of the dynamic processes of vegetation growth. The above models were constructed by Matlab r2021a.

RF algorithm, employing decision trees as base learners (Beriman, 2001), constructs multiple trees in parallel by randomly selecting attributes. The prediction results of all decision trees are averaged to obtain the final regression modeling result of the entire random forest. Due to its random sampling and feature generation methods in decision trees, RF can improve the prediction accuracy of the model without significantly increasing computational complexity. The key parameters in RF include the number of trees and the number of nodes. After repeated debugging and optimization, the number of trees was determined to be 500, and the minimum number of samples for leaf nodes was set to 8 in this study.

XGBoost, an enhanced gradient boosting algorithm, combines multiple weak classifiers into a robust classifier (Chen and Guestrin, 2016). By separating the selection of the loss function from the optimization of the modeling algorithm and the selection of parameters, the algorithm can adaptively choose the appropriate loss function based on specific requirements or objectives, thereby enhancing the algorithm’s applicability (Dhaliwal et al., 2018). XGBoost intergrates weak classifiers and enabling flexible loss function selection to enhance the modeling capability and overall performance. The primary settings for the kernel parameters were as follows: learning rate was set to 0.5, the maximum depth of tree was set to 1, the gamma was set to 0.01, the regularization parameters alpha and lambda were set to 0.02 and 0.1, respectively. Additionally, the subsampling method, subsample was set to 0.3, and colsample_bytree was set to 0.5.

SVM was chosen for small sample learning (Zhang et al., 2021a), utilizing the Radial Basis Function (RBF) kernel function. Its fundamental idea is to find an optimal hyperplane that minimizes the error between training sample points and the hyperplane. The optimal kernel parameters (g) and regularization parameter (c) were determined through adjustment and optimization. BPNN possesses strong fault tolerance and adaptive learning capabilities. It consists of input layers, hidden layers, and an output layer (Panda et al., 2010). By continuously adjusting the number of neurons in the hidden layers, the data is iteratively trained to obtain an optimal model. In this study, the number of hidden layers and the number of nodes were determined to be 1, respectively.

CNNs are capable of unsupervised feature learning, demonstrating remarkable performance in automated feature acquisition.The model architecture comprises convolutional layers, pooling layers, batch normalization layers, fully connected layers, dropout layers, and a regression layer (Lee et al., 2015). In this study, the size of the convolutional kernel was set to half the number of input variables. The Rectified Linear Unit (ReLU) activation function was used to accelerate the convergence speed of the model. During the training process, the dropout layer was employed with a dropout rate of 20% to enhance the generalization ability of the model and prevent overfitting. The Stochastic Gradient Descent with Momentum (SGDM) algorithm was utilized to optimize the weights of the model, and the initial learning rate was set to 0.01.

LSTM algorithm was built upon the foundation of recurrent neural networks, which introduces a gating mechanism to control the path of information transmission (Hochreiter and Schmidhuber, 1997). It utilizes input gates, forget gates, and output gates to dynamically adjust the weights of self-recurrent. In this way, when the model parameters are fixed, the integration scale at different times can change dynamically, so as to effectively address the challenges of gradient explosion or disappearance of simple recursive neural network. The main parameter settings were consistent with CNN.

#### 2.4.2 Accuracy evaluation

The samples were divided into calibration and validation sets using the sample set partitioning based on joint x-y distance (SPXY)(Galvao et al., 2005), which is based on the Kennard-Stone algorithm. This method considers both the feature variable (x) and the target variable (y) when selecting the data, aiming to determine a sub-feature space that maximally represents the original data space. It achieves this by calculating the relative Euclidean distance within the data space. By applying SPXY, the selected samples are more uniformly distributed and reasonably divided, providing a more comprehensive representation of vegetation conditions within the study area. The use of this approach may improve the repeatability and generalizability of the study.


(5) $$d_{xy}(p,q)=\frac{d_{x}(p,q)}{\underset{p,q\in \left[1,N\right]}{\max}d_{x}(p,q)}+\frac{d_{y}(p,q)}{\underset{p,q\in \left[1,N\right]}{\max}d_{y}(p,q)}\;,\left(\text{p,q}\in \left[1,N\right]\right)$$

(6) $$d_{x}(p,q)=\sqrt{\sum_{j=1}^{J}{\left[x_{p}\left(j\right)-x_{q}\left(j\right)\right]}^{2}}\;,\left(\text{p,q}\in \left[1,N\right]\right)$$

(7) $$d_{y}(p,q)=\sqrt{{\left({\text{y}}_{p}{\text{- y}}_{q}\right)}^{2}}\;,\left(\text{p,q}\in \left[1,N\right]\right)$$

where *d_xy_ (p,q)* represents the Euclidean distance of two spaces considered, *d_x_(p,q)* represents the Euclidean distance of two samples in x space (feature space), *d_y_(p,q)* represents the Euclidean distance of two samples in y space (target space), max *d_x_(p,q)* and max *d_y_(p,q)* denotes the maximum Euclidean distance of p and q in x and y space respectively. *N* is the total number of samples; *J* is the number of feature spaces. *x_p_(j)* and *x_q_(j)* respectively represent the values of p and q samples on the j-th feature. where *y_p_* and *y_q_* are the Euclidean distances of the two samples in y space.

The calibration dataset for estimating winter wheat LAI values was constructed using 70% of the sample data, while the remaining 30% served as the validation dataset for evaluation. The accuracy of the model was assessed using various performance metrics, including the Coefficient of Determination (R^2^), Root Mean Square Error (RMSE), and Ratio of Performance to Standard Deviate (RPD). For the evaluation indexes mentioned above, a higher R^2^ value indicates a better prediction effect, while a smaller RMSE indicates a more accurate model. In terms of RPD, it is generally considered that RPD<1.4 indicates an unreliable model, 1.4< RPD< 2.0 suggests a moderate reliability, and model has a higher level of reliability if RPD > 2.0. These metrics provide quantitative measures to assess the accuracy and reliability of the model in estimating the winter wheat LAI.


(8) $$R^{2}=\frac{\sum_{i=1}^{n}{\left(x_{i}-\bar{x}\right)}^{2}{\left(y_{i}-\bar{y}\right)}^{2}}{\sum_{i=1}^{n}{\left(x_{i}-\bar{x}\right)}^{2}\sum_{i=1}^{n}{\left(y_{i}-\bar{y}\right)}^{2}}$$

(9) $$RMSE=\sqrt{\frac{\sum_{i=1}^{n}{\left(y_{i}-\bar{y}\right)}^{2}}{n}}$$

(10) $$RPD=\frac{SD(x_{\text{i}})}{RMSE}$$

where *x_i_* and $\bar{x}$ represent the measured value and its mean value, *y_i_* and $\bar{y}$ are the predicted value and its mean value of each sample, respectively. *n* indicates the number of samples in the calibration set or validation set. *SD* is the standard deviation of the measured value of the sample.

## 3 Results

### 3.1 Correlation between vegetation indices and winter wheat LAI

**Figure 4:**
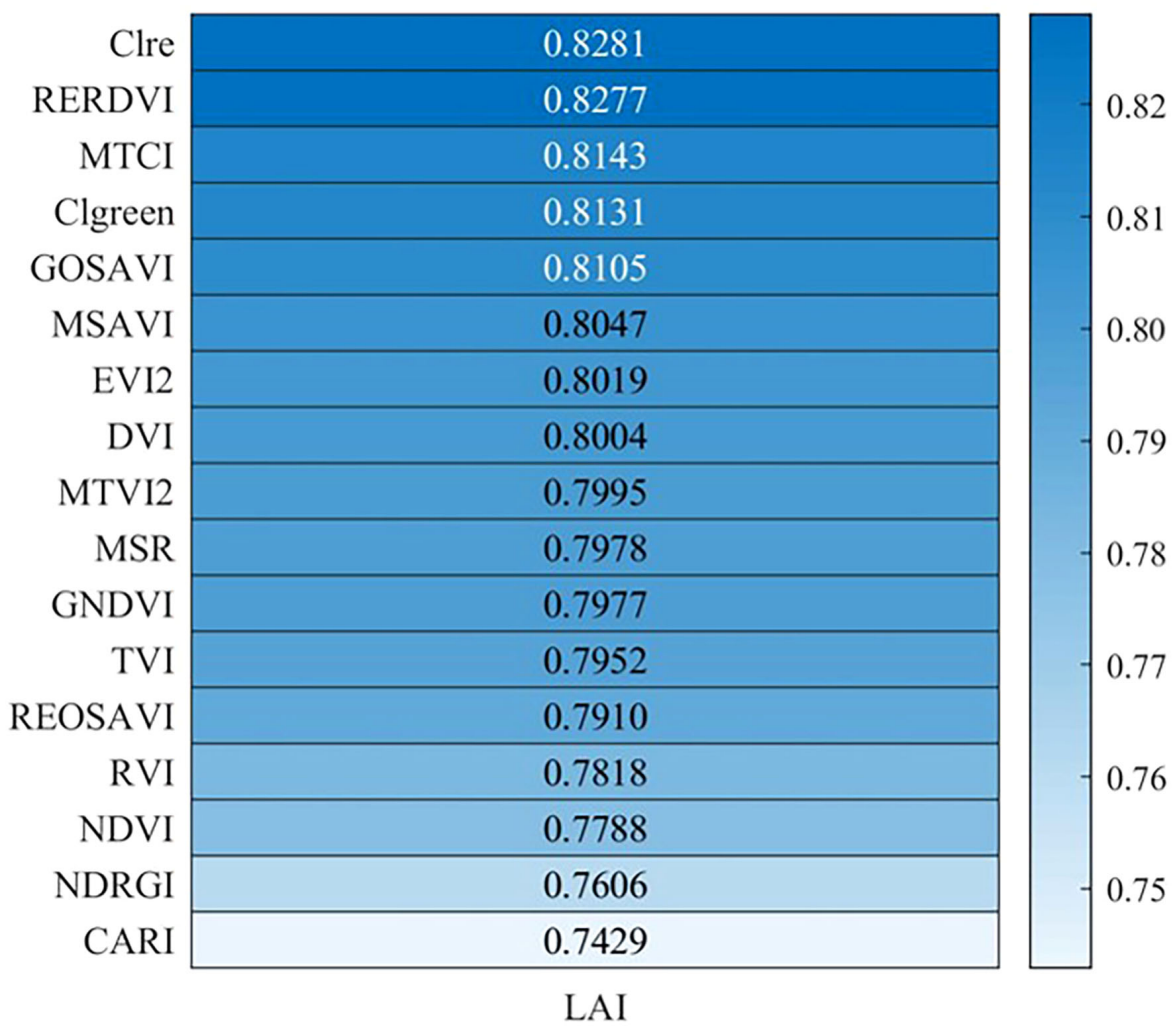
Correlation coefficients between winter wheat LAI and vegetation indices (P<0.01).

The correlation between the selected 17 vegetation indices and the measured LAI in the field was analyzed. The Pearson correlation coefficient is shown in **Figure 4**. The results revealed that all vegetation indices showed a correlation coefficient with LAI above 0.74, indicating a highly significant relationship (p<0.01). Among the vegetation indices, Clre exhibited the strongest correlation with LAI, with a correlation coefficient of 0.83, while CARI exhibited the weakest correlation, with a correlation coefficient of 0.74. Based on these findings, vegetation indices with correlation coefficients greater than 0.80 were selected as independent variables. The selected VI, ranked in descending order of correlation coefficient, were Clre, RERDVI, MTCI, Clgreen, GOSAVI, MSAVI, EVI2 and DVI, with corresponding correlation coefficients of 0.83, 0.83, 0.81, 0.81, 0.81, 0.80, 0.80, and 0.80, respectively.

### 3.2 Correlation between texture features and winter wheat LAI

**Figure 5:**
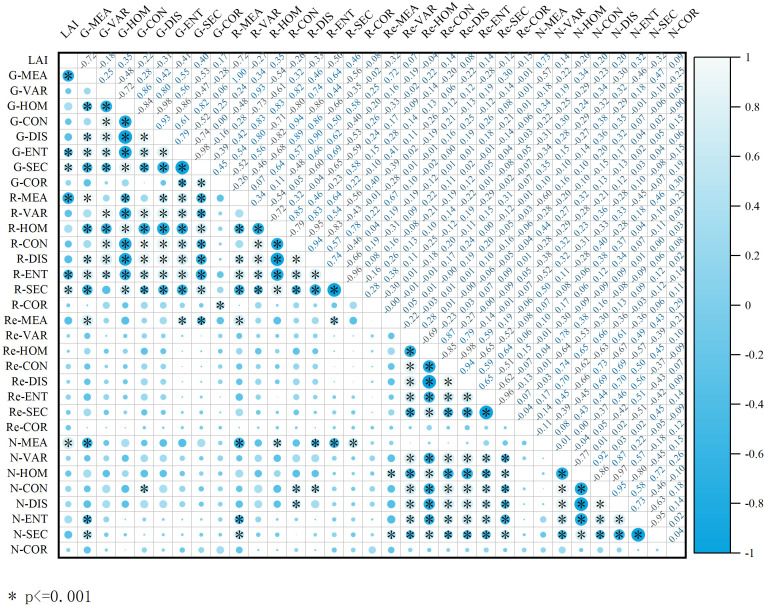
Correlation coefficients between winter wheat LAI and texture features (* represents P ≤ 0.001).

Based on the correlation analysis between the selected texture features and LAI, it is evident from **Figure 5** that more than half of the texture features exhibited a relatively low correlation with LAI. Only a small subset of texture features demonstrated a high correlation with LAI. Specifically, the MEA in the green band, near-infrared band, and red band exhibited correlation coefficients of 0.72, 0.73, and 0.72, respectively, indicating a strong correlation (P<0.01). However, for other texture features showing a highly significant correlation, the absolute values of the correlation coefficients generally ranged from 0.18 to 0.50. Given the relatively weak correlation between texture features and LAI, their utility for accurate LAI prediction was limited. Consequently, this study employed some texture indices composed of texture features from different bands, specifically NDTI, DTI and RTI.

**Figure 6:**
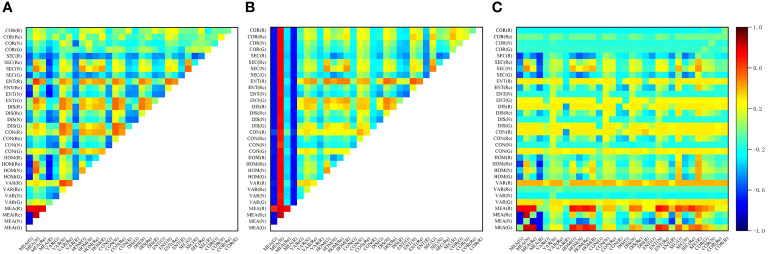
Correlation coefficients between winter wheat LAI and texture indices: **(A)** NDTI; **(B)** DTI; and **(C)** RTI. The abscissa and ordinate represent the correlation coefficient between the texture index and LAI of the corresponding two texture features after normalization, difference and ratio operations.

**Figure 7:**
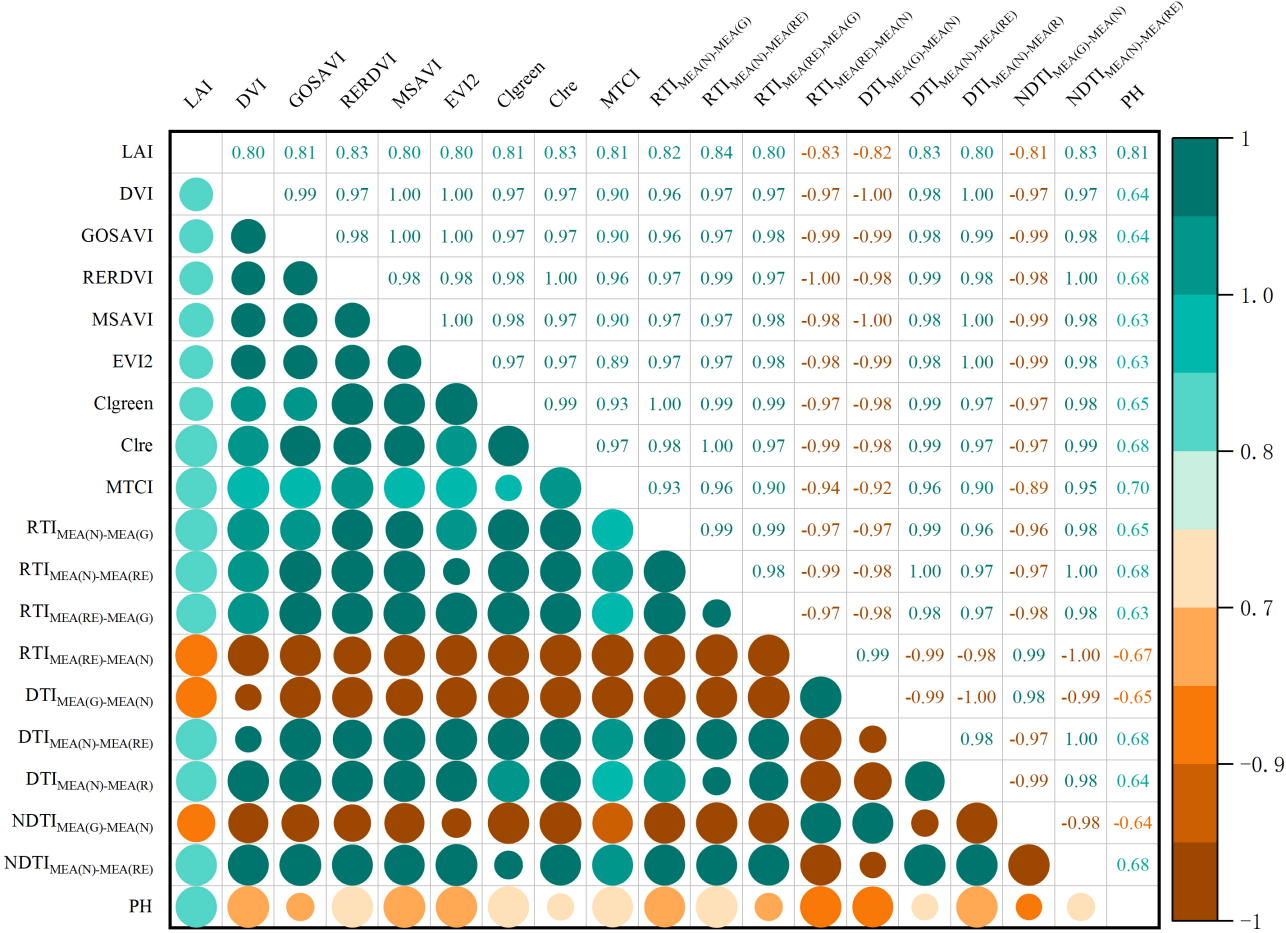
Correlation coefficients between winter wheat LAI and input features (VI, TI, PH).

The correlation analysis between the texture indices and LAI showed that by combining the texture features, the overall correlation between the texture features and LAI was significantly improved. **Figure 6** shows a high correlation between the combination of MEA for each band and LAI. Similarly, texture indices with a correlation coefficient greater than 0.80 were selected as independent variables. The correlations, listed from highest to lowest, were as follows: the ratio and normalized difference between the MEA of the near-infrared and red edge bands (RTI_MEA(N)-MEA(RE)_, NDTI_MEA(N)- MEA(RE)_), the ratio and difference between the MEA of the red edge and near-infrared bands (RTI_MEA(RE)-MEA(N)_, DTI_MEA(N)-MEA(RE)_), the ratio, difference and normalized value between the MEA of the near-infrared and green bands (RTI _MEA(N)-MEA(G)_, DTI_MEA(G)-MEA(N)_, NDTI_MEA(G)-MEA(N)_), the difference between the MEA of the near-infrared and red bands (DTI_MEA(N)-MEA(R)_), and the ratio between the MEA of the red edge and green bands (RTI_MEA(RE)-MEA(G)_). The correlation coefficients for these TI were 0.84, 0.83, -0.83, 0.83, -0.82, 0.82, -0.81, 0.80, and 0.80, respectively. **Figure 7** shows the correlation coefficients between winter wheat LAI and input features, including VI, TI and PH.

### 3.3 Winter wheat LAI estimation

**Figure 8:**
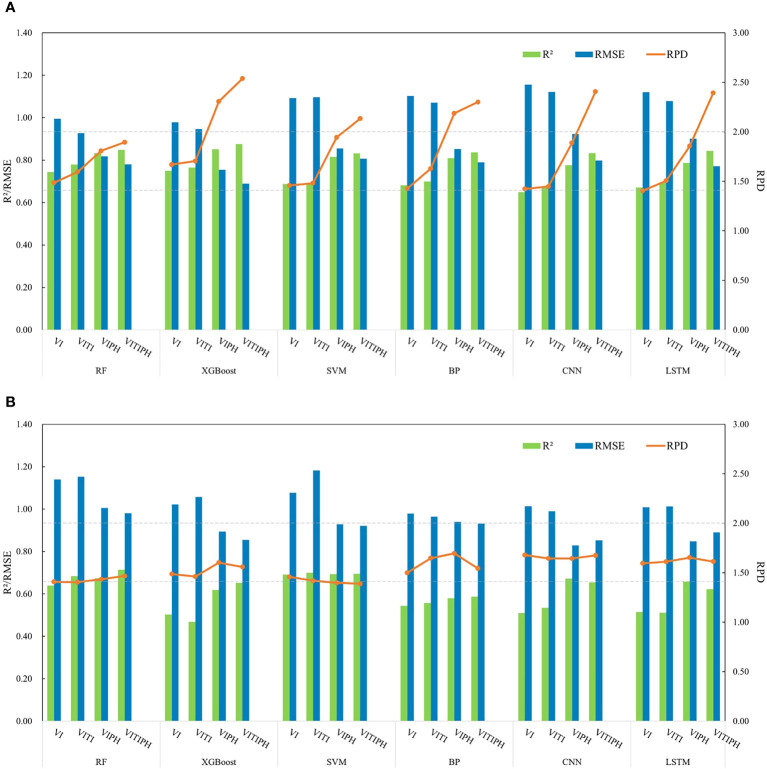
Accuracy of winter wheat LAI estimation based on different algorithms and input variables. **(A)** calibration dataset; **(B)** validation dataset.

Using four combinations of VI, VITI, VIPH, and VITIPH as input data, six regression algorithms including RF, XGBoost, SVM, BPNN, CNN, and LSTM were employed to estimate LAI for winter wheat at jointing stage, and their accuracy was evaluated. The results are shown in **Figure 8**.

#### 3.3.1 LAI estimation based on different features

In the RF model, the accuracy of the LAI estimation model constructed using VITI and VIPH input variables was improved compared to the model constructed using VI (R^2^ = 0.74, RMSE=0.99, RPD=1.49). The R^2^, RMSE and RPD of the input VITI modeling set were 0.78, 0.93 and 1.60 respectively, and the R^2^, RMSE and RPD of the validation set were 0.68, 1.15 and 1.40 respectively. For the VIPH modeling set, R^2^, RMSE, and RPD were 0.83, 0.82, and 1.81, respectively, while the validation set yielded R^2^ of 0.67, RMSE of 1.01, and RPD of 1.43. And the model constructed using VITIPH demonstrated the highest accuracy (R^2^ = 0.85, RMSE=0.78, RPD=1.89). The XGBoost and RF algorithms exhibited similar model performance. Further analysis was conducted using the XGBoost method, which showed high overall accuracy. The calibration set constructed by inputting VITIPH showed the best performance among all regression models, achieving the highest R^2^ (R^2^ = 0.88), the lowest RMSE (RMSE=0.69), and an RPD greater than 2 (RPD=2.54).

Based on the SVM model, the models constructed using VI (R^2^ = 0.69, RMSE=1.09, RPD=1.46) and VITI (R^2^ = 0.69, RMSE=1.10, RPD=1.48) as input feature variables showed similar results in terms of model accuracy evaluation metrics on the calibration and validation sets. The model constructed by introducing PH as the input variable (VIPH, VITIPH) showed better effect than VI and VITI. The R^2^, RMSE and RPD of calibration set constructed by VIPH were 0.82, 0.85 and 1.94 respectively, and the R^2^, RMSE and RPD of validation set were 0.69, 0.93 and 1.40 respectively. The effect of the model constructed by VITIPH was the best. The R^2^ and RPD of the modeling set increased to 0.83 and 2.13 respectively, and the RMSE decreased to 0.81.

The models constructed using the BPNN, CNN, and LSTM neural network algorithms also demonstrated positive predictive performance in estimating winter wheat LAI (all RPD > 1.4). The R^2^ of the calibration set were all above 0.64, and the R^2^ of the validation set were all above 0.51. The RMSE ranged from 0.77 to 1.16. All the three models showed that the model based on the combination of characteristic input variables of VITI and VIPH performed better than the regression model with VI input, especially the inclusion of the PH further enhanced the performance of the model. In the calibration set, the BPNN model showed an increase in R^2^ from 0.6818 to 0.8096, a decrease in RMSE from 1.10 to 0.85, and an increase in RPD from 1.43 to 2.19. Similarly, the CNN model exhibited an increase in R^2^ from 0.65 to 0.78 and a decrease in RMSE from 1.16 to 0.92 in the calibration set. The modeling dataset of the LSTM model also had an increase in R^2^ from 0.67 to 0.79, and in the validation dataset, with an increase in R^2^ from 0.52 to 0.66. Meanwhile, the best model performance was achieved when using the VITIPH as input variables. In the BPNN model, the R^2^, RMSE and RPD for the modeling dataset were 0.84, 0.79, and 2.30, respectively. In the CNN model, the R^2^, RMSE, and RPD for the modeling dataset were 0.83, 0.80 and 2.41, respectively. In the LSTM model, the R^2^, RMSE, and RPD for the modeling dataset were 0.84, 0.77 and 2.39, respectively, while for the validation dataset, the R^2^, RMSE and RPD were 0.62, 0.89 and 1.61, respectively.

Overall, the models constructed using different combinations of features demonstrated better performance compared to those relying solely on VI input, with the following order of accuracy: VI<VITI<VIPH<VITIPH. This indicated that introducing other feature variables can effectively improve the accuracy of the winter wheat LAI estimation model. Notably, the incorporation of PH as a variable significantly enhanced the model’s ability to estimate LAI. As an essential plant parameter, PH exhibited a strong correlation with LAI, making its inclusion in model construction crucial for achieving more accurate LAI estimation.

#### 3.3.2 Combining VI, TI, and PH to estimate LAI using different algorithms

**Figure 9:**
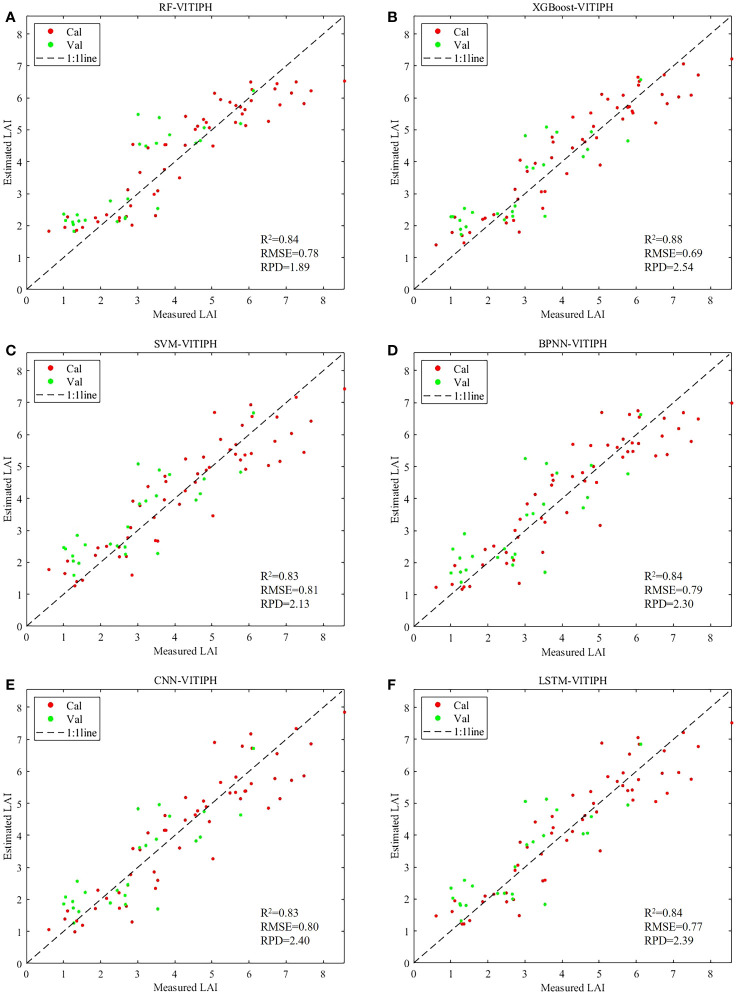
Scatter diagram of LAI estimation results of winter wheat with six algorithms using VITIPH as input variables. **(A)** RF-VITIPH; **(B)** XGBoost-VITIPH; **(C)** SVM-VITIPH; **(D)** BPNN-VITIPH; **(E)** CNN –VITIPH; **(F)** LSTM -VITIPH. The calibration set and validation set are represented in red and green point respectively.

The scatter distribution of the measured and estimated LAI values obtained from the VITIPH input, using six algorithms, is presented in **Figure 9**. It was evident that the fitted distribution of the winter wheat LAI estimation, constructed based on the XGBoost algorithm, closely approximated a 1:1 relationship, indicating its superior predictive capability. However, some issues remained, such as overestimation of low values and underestimation of high values. Notably, the SVM, RF, and XGBoost models exhibited robust stability when compared to the BPNN, CNN, and LSTM neural network models. This is primarily attributable to the superior adaptability of SVM, RF, and XGBoost to small-sample datasets. Even in situations with limited data volume, they can provide relatively accurate estimates. Furthermore, these algorithms exhibit robustness to outliers and noise within the data. This resilience stems from the fundamental principles of these algorithms; for instance, SVM demonstrates enhanced tolerance to outliers through the concept of support vectors, while RF and XGBoost effectively mitigate the sensitivity of individual decision trees to noise by employing ensemble learning techniques. Moreover, SVM, RF, and XGBoost models possess a relatively parsimonious set of hyperparameters compared to neural network models. These attributes contribute to the stability and reliability of the models during the training process, thereby manifesting superior performance in our research.

Moreover, it can be observed from **Figures 8**, **9** that a consistent trend occurred when comparing the LAI estimation using various regression algorithms. Combined vegetation indices and texture indices with plant height, the calibration set R^2^ of the six algorithms was greater than 0.8, indicating that the accuracy of LAI estimation by machine learning and deep learning regression algorithm was high.

### 3.4 Winter wheat LAI Inversion map

**Figure 10:**
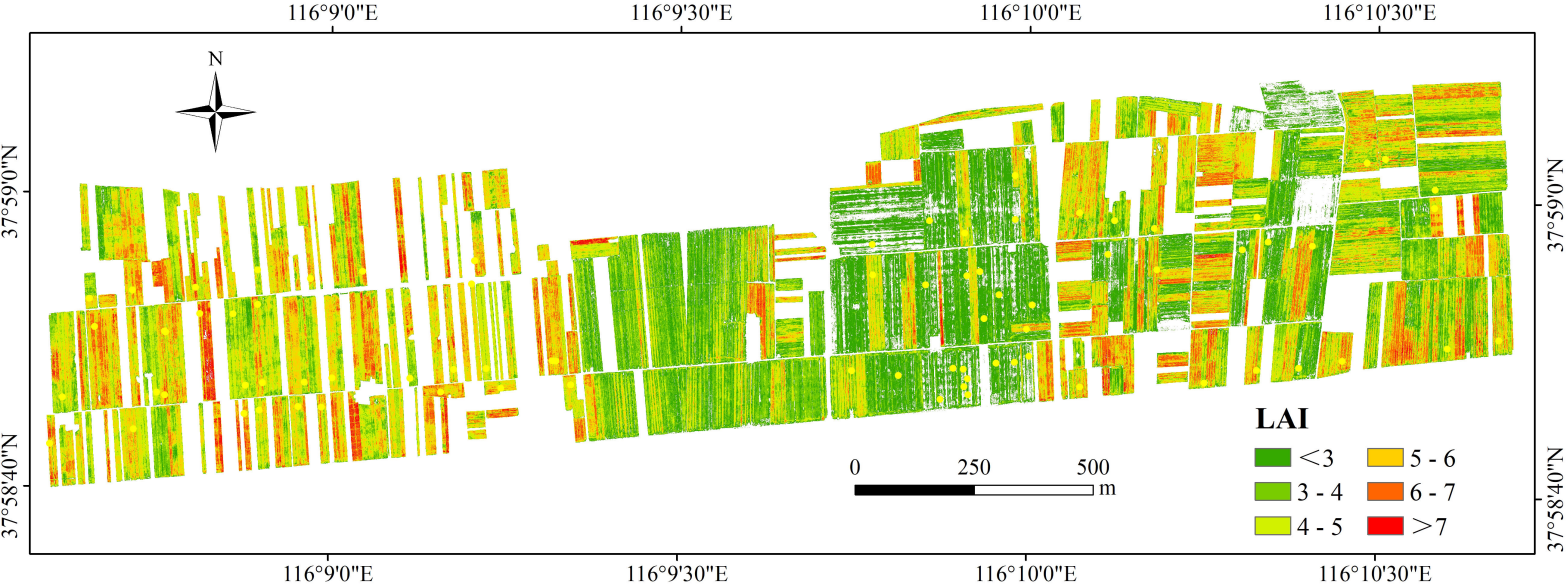
Spatial distribution of winter wheat LAI estimation results based on optimal method in the study area.

The XGBoost model, which combined VI, TI, and PH, achieved the most accurate prediction of winter wheat LAI. This model was utilized to generate a spatial distribution map of winter wheat LAI inversion in the study area, LAI values range from 0.96 to 8.86, as depicted in **Figure 10**. By extrapolating LAI from the pixel scale to the regional scale, remote sensing monitoring of winter wheat LAI at a broader scale was achieved. This approach facilitated a more comprehensive understanding of the growth status and spatial distribution patterns of winter wheat in the region. The findings will provide valuable information for real-time monitoring of wheat growth and development, as well as the formulation of customized fertilization prescriptions and other agricultural production management and decision-making processes.

For example, the plant water use efficiency can be evaluated through the further analysis of LAI inversion map.This aids in the rational development of irrigation plans, ensuring that plants receive adequate water and enhancing the overall water resource utilization efficiency in agricultural fields. Simultaneously, the leaf area of a plant is associated with nutrient absorption. Agricultural practitioners can leverage LAI inversion maps to precisely understand the nutrient requirements of plants, thereby optimizing fertilizer plans and improving fertilizer efficiency. Furthermore, agricultural decision-makers can utilize LAI inversion maps for early detection of diseases and pests, enabling the implementation of preventive or curative measures to mitigate the adverse impact of plant diseases on yield. Additionally, LAI inversion maps can be employed for preliminary estimations of crop yields.

## 4 Discussion

The main objective of this study was to explore the potential of using ML/DL to improve the accuracy of winter wheat LAI estimation by combining VI, TI and PH based on UAV multispectral image.

The study revealed a robust correlation between vegetation indices and LAI for winter wheat during the jointing period. The selected 17 vegetation indices (RVI, DVI, NDVI, GNDVI, EVI2, Clgreen, MSR, MSAVI, GOSAVI, REOSAVI, RERDVI, Clre, CARI, NGRDI, TVI, MTVI2, MTCI) in this study demonstrated correlations exceeding 0.74 with LAI, with p-values lower than 0.01, signifying a highly significant relationship. Notably, indices like Clre, RERDVI, MTCI, and Clgreen, founded on the ratio of near-infrared to visible light bands, provide insights into chlorophyll content and photosynthetic activity. Given that LAI of winter wheat characterizes the total area of the leaves, and the chlorophyll content in the leaves is related to LAI in some cases. During the jointing stage, winter wheat exhibits higher chlorophyll content and more vigorous photosynthetic activity, fostering in a strong correlation between these indices and LAI. Moreover, certain indices depict alterations in vegetation structure and coverage, such as NDVI, MSAVI, and EVI2. As winter wheat grows, the plant’s structure progressively develops, leading to an increase in leaf number and density, alongside expanding vegetation coverage, ultimately resulting in elevated LAI. These indices sensitively capture variations in vegetation structure and coverage, thereby demonstrating a significant positive correlation with LAI. Indices such as EVI2 and DVI have been corrected for soil background and atmospheric effects, effectively reducing interference with vegetation reflectance and providing more precise information regarding wheat LAI. Consequently, these indices exhibit a significant correlation with LAI. The integration of these diverse vegetation indices contributes to a comprehensive understanding of LAI dynamics for winter wheat during the jointing period, enhancing the accuracy of LAI estimation through remote sensing approaches.

Nevertheless, the majority of texture features exhibited a weak correlation with LAI. In order to address this limitation, combining multiple texture features to create a new texture index can integrate diverse and comprehensive texture information. This approach mitigates the influence of soil, terrain, and shadow backgrounds while accentuating pertinent features (Hang et al., 2021). It is worth noting that the extraction of texture features is often susceptible to image noise, variations in illumination, and other interfering factors, which may lead to the instability of the results of a single texture feature. By employing combination operations, random noise within individual features can be eliminated or reduced, while uncertainties stemming from changes in illumination and other factors can be minimized. This contributes to enhancing the stability and reliability of the correlation between texture features and LAI. The NDTI, DTI and RTI formed by the combination of MEA of each band have high correlation with LAI. The mean value of texture measurement includes the average value of the target and background in the moving window, which can smooth the image and minimize background interference (Wang et al., 2022b). Additionally, green vegetation absorbs most visible light in the red edge band, and in the near-infrared band, the diffuse reflection of the canopy structure leads to a higher reflectance in the near-infrared region (Yu et al., 2020). The difference, ratio, and normalized difference between the near-infrared and red edge bands can enhance the difference in light absorption and reflection of vegetation, thus better reflecting the canopy structure of green vegetation. This further reinforces the capability of texture information in representing LAI, aligning with the findings of Zhang (Zhang et al., 2022a). Through the comparison of six regression algorithms, this study identified that integrating VI and TI enhanced the performance of the LAI estimation model.

**Figure 11:**
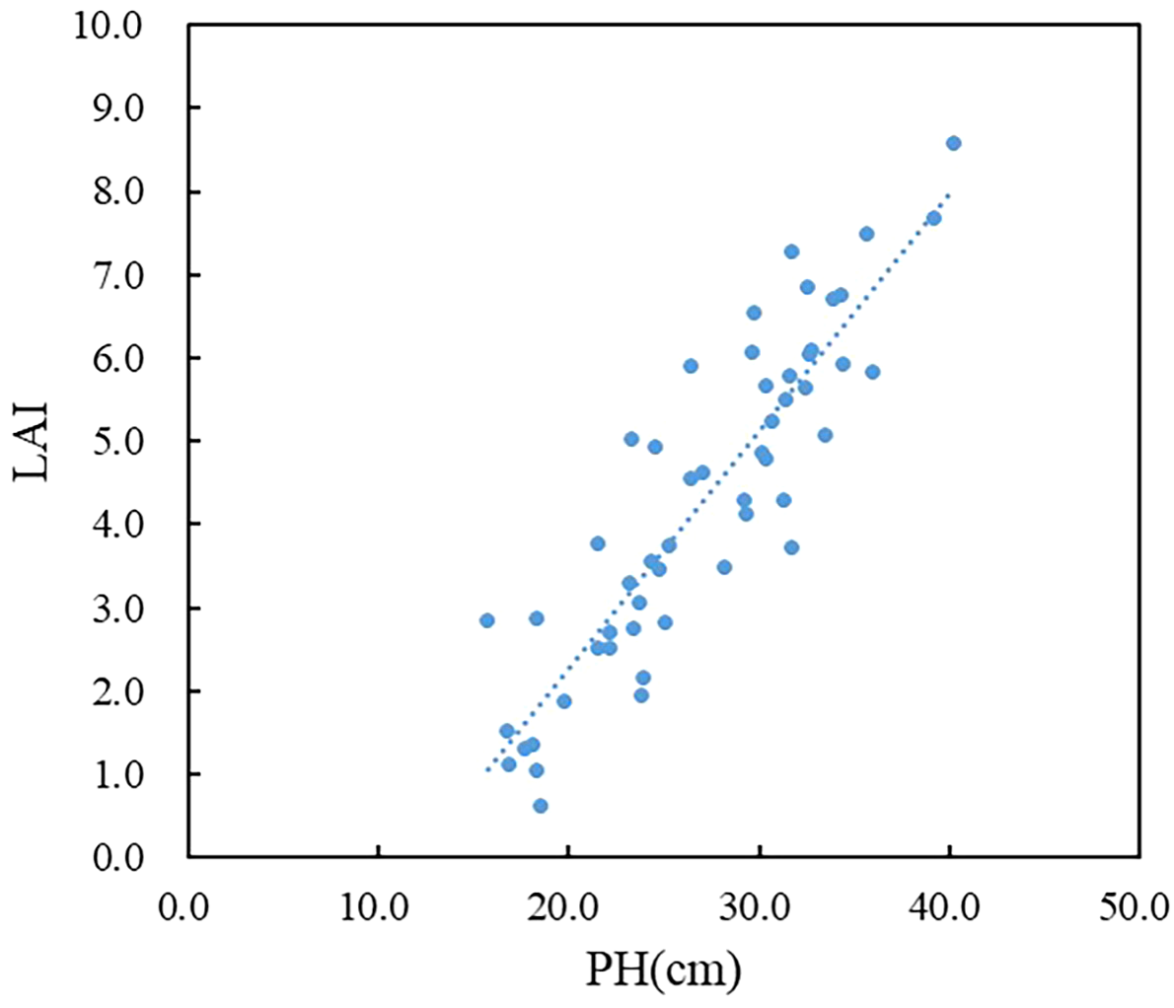
Scatter plot between LAI and PH.

However, solely combining TI does not effectively address the saturation issue of VI in high-density canopies. On the other hand, incorporating plant structure information, such as PH, proves more beneficial in addressing or improving this concern. Analysis of **Figure 11** reveals a clear linear positive correlation between PH and LAI during the jointing stage of winter wheat, indicating that LAI progressively increases with the growth of wheat plant height. This is attributed to the fact that an increase in wheat plant height is often accompanied by a corresponding increase in leaf area. The expanded leaf area enables the plant to absorb and utilize more light energy, which facilitates enhanced photosynthesis, increases organic matter production, and promotes overall plant growth and development. It is also used to form plant organs such as roots, stems and ears, which reacts on the increase of plant height. Yuan et al. (Yuan et al., 2013) have demonstrated a significant positive correlation between vegetation canopy height and LAI. Accordingly, this study incorporated the structural information of PH extracted from UAV images in the construction of the winter wheat LAI estimation model.Furthermore, it is worth noting that this paper achieved a satisfactory level of accuracy in predicting plant height based on UAV-derived data, with an R^2^ of 0.86 and an RMSE of 2.07cm between predicted and measured values.

Studies have indicated that when constructing a LAI estimation model with a single type of feature input as a variable, the model constructed by texture feature is not as stable as the VI and is not suitable for estimating crop LAI as an independent feature variable (Zhang et al., 2021b). Therefore, this paper only considered the VI as a single type of feature variable to construct the model. Simultaneously, three types of multi-feature fusion combinations were considered (VITI, VIPH, VITIPH). The results showed that, compared to a single type of variable, the winter wheat LAI estimation model constructed by combining VI and TI with PH exhibited the highest accuracy. Furthermore, the combination of VI and PH in the model construction exhibited significantly higher accuracy compared to models based solely on VI or the combination of VI and TI in estimating winter wheat LAI. This highlighted the feasibility of integrating plant height and spectral information in LAI estimation, leading to an improvement in model accuracy and alleviating the issue of spectral saturation. For future studies, it is recommended to consider the inclusion of other crop vertical structural features during model construction, which may further enhance LAI estimation accuracy and provide comprehensive insights into the growth dynamics of winter wheat.

Analyzing the estimated results, it can be seen that all six algorithms used in this paper achieved strong ability to estimate winter wheat LAI. The RF model constructed using VITIPH obtained the R^2^ was 0.85, RMSE was 0.78, and RPD was 1.90. The R^2^, RMSE and RPD of calibration set constructed by SVM using VITIPH were 0.83, 0.81 and 2.13 respectively. The models constructed using the BPNN, CNN, and LSTM neural network algorithms also demonstrated positive predictive performance in estimating winter wheat LAI (all RPD > 1.4). It is notable that the model based on the XGBoost algorithm demonstrated the highest performance among all the models tested, achieving the highest R^2^ (R^2^ = 0.88), the lowest RMSE (RMSE=0.69), and an RPD greater than 2 (RPD=2.54). By comprehensively comparing the R^2^, RMSE and RPD of the correction set and the verification set of the six algorithms, it was found that the six evaluation indexes of the model performed well as a whole, so XGBoost was selected as the most optimal model. The advantage of this algorithm is that it is an ensemble learning algorithm, which constructs a strong classifier by combining multiple weak classifiers (decision trees) (Chen and Guestrin, 2016). And ensemble learning enables the combination of predictions from multiple models, reducing individual model biases and variances, and improving the overall generalization ability of the model. This feature allows XGBoost to effectively capture the intricate relationship between LAI and the input features, thereby enhancing the performance of the model. Additionally, XGBoost employs an optimized second-order Taylor expansion, enabling it to accurately estimate complex nonlinear datasets and achieve superior results. Additionally, the SPXY algorithm was used to screen the calibration samples and validation samples before constructing the model in this paper, which considered the distance between different feature variables and the distance between target variables at the same time. By doing so, the algorithm ensures a more even distribution and reduces the differences between the calibration and validation sets, thus improving the accuracy of the model. Researches have proved that machine learning and deep learning algorithms combined with remote sensing features had a good effect on LAI estimation of winter wheat (Li et al., 2021; Zhang et al., 2021b). In this study, the performance of the CNN and LSTM algorithms did not surpass expectations, potentially attributed to the limited sample size. It might have hindered the ability of deep learning to fully leverage its advantages, resulting in a decline in model performance. Increasing the sample size has various positive impacts on the performance of deep learning models. Firstly, a larger sample size enhances the model’s generalization ability, allowing it to better adapt to a wide range of data distributions, thus exhibiting more robust performance when faced with new, unseen data. Additionally, the increase in sample size helps mitigate the risk of overfitting, making the model’s performance on the test set more reliable.Ultimately, a greater sample size provides more accurate model evaluations, offering a more reliable basis for the objective assessment of model performance. In general, the deep learning has advantages in processing complex, high-dimensional and large-scale data, and can automatically learn and extract features (Nevavuori et al., 2019). However, due to its large demand for data and computing resources, high requirements for interpretability and comprehensibility, complex hyper-parameters selection and optimization, and sensitivity to data quality and noise, it may be limited in these aspects when processing some tasks, which is relatively inferior to machine learning algorithms. Therefore, selecting an appropriate algorithm needs to comprehensively consider the task requirements, data characteristics and resource constraints.

This paper specifically focused on the estimation of winter wheat LAI during the jointing stage. Whether the accuracy of multiple features combination modeling is higher than that of single type of VI modeling in the whole growth cycle remains to be further studied. Additionally, it is important to examine whether the XGBoost algorithm maintains the highest LAI estimation accuracy across the entire growth period of winter wheat. After that, the data collection of the whole growth cycle of crops should be carried out to more comprehensively grasp the growth state of winter wheat. At the same time, the universality and applicability of the findings should be further studied in different regions.

The flight altitude of UAV is often closely linked to ground resolution. The extraction of texture features at varying ground resolutions has a discernible impact on the monitoring of Wheat LAI. Estimating LAI typically necessitates the consideration of vegetation texture information, encompassing leaf arrangement, size, and morphology. When the flight altitude is set at 9 cm, UAV sensors can capture finer details of ground vegetation, thus providing a richer source of texture information. This, in turn, contributes to a more precise characterization of vegetation structure and distribution. Given that this study was conducted with specific flight altitude and spatial resolution settings tailored for agricultural fertilization decisions, future research may require experiments involving different flight altitudes to validate the methodologies and outcomes delineated in this paper.

The method utilized in this study may also be extended to satellite data, such as WorldView-3 satellite, ZY-3 satellite, and others. High spatial resolution satellite remote sensing offers rich spectral and texture information, while plant height can be extracted through multi-temporal stereo image pairs. However, the critical growth and development period of crops is often relatively short, emphasizing the necessity of obtaining relevant satellite data within a specific time window. Challenges may arise when the satellite’s coverage and revisit cycle fail to meet the data requirements during this crucial period. Additionally, the quality of satellite imagery becomes difficult to control due to adverse weather conditions, such as clouds and rain, which can lead to the loss of significant monitoring opportunities. In future research endeavors, satellite data retains its potential for monitoring crop growth parameters in lager areas, possibly surmounting the previously mentioned challenges through techniques like image fusion.

According to this research, it was known that UAV multispectral data played a pivotal role in estimating LAI. However, it is crucial to recognize and address certain limitations associated with the utilization of multispectral data. This constraint arises from the inherently restricted spectral resolution of multispectral data, wherein the UAV-mounted multispectral sensor captures information within a limited set of pre-defined bands. This limitation has the potential to hinder the precise characterization of subtle variations in vegetation properties. In contrast, hyperspectral data, characterized by capturing an extensive range of contiguous narrow bands across the electromagnetic spectrum (Tao et al., 2020), offer increased spectral resolution. The augmentation in resolution contributes to a more accurate characterization of vegetation attributes, potentially enhancing the accuracy of LAI estimation. Additionally, LiDAR data, capable of capturing three-dimensional structural information of vegetation, serves as a complementary dataset to spectral data (Luo et al., 2018). The enhancement of LAI estimation is primarily achieved by considering the vertical structure of vegetation. Therefore, it is worthwhile to consider the utilization of hyperspectral or LiDAR data for vegetation LAI estimation in future research endeavours.

In this study, winter wheat LAI monitoring based on UAV multiple image provides the underlying data for wheat production. The future work will apply this approach to different wheat growth periods under various production management practices, and explore the applicability in different regions and scales.

## 5 Conclusion

This study utilized UAV multispectral images to extract spectral and texture information, while incorporating plant height information for LAI estimation. The comparative analysis was conducted to assess the efficacy of four machine learning and two deep learning regression algorithms in estimating the LAI of winter wheat. The main conclusions were as follows:

1. At the jointing stage of winter wheat, the PH derived from UAV images played an important role in LAI estimation, which can improve the estimation accuracy of winter LAI.
2. The combination of texture features can significantly improve the correlation between texture features and LAI, especially the combination of the MEA of each band had high correlation with wheat LAI. Comparing VI, VITI, VIPH and VITIPH as input variables, it was found that the ability of combining multiple features to estimate LAI of winter wheat was better than the estimation model constructed by only inputting VI, and the fusion of three kinds of features involved in the construction of the estimation model was the best.
3. The machine learning and deep learning algorithms were shown promising results in accurately estimating winter wheat LAI using UAV remote sensing data. The RF, XGBoost and SVM model constructed using VITIPH obtained the R^2^ values of 0.85, 0.88 and 0.83, while the R^2^ of BPNN, CNN, and LSTM were 0.81, 0.78 and 0.79, respectively. It is notable that the model based on the XGBoost algorithm demonstrated the highest performance among all the models tested.

The research results demonstrate that UAV data and advanced algorithms will provide technical support for the rapid and nondestructive estimation of winter wheat LAI and help to formulate variable rate fertilization prescription of agricultural machinery. This research provides a valuable framework for optimizing agricultural practices, underscoring their potential of leveraging advanced technologies for precision agricultural and making significant contributions to sustainable farming.

## Data availability statement

The original contributions presented in the study are included in the article/supplementary material, further inquiries can be directed to the corresponding authors.

## Author contributions

MZ: Conceptualization, Data curation, Formal Analysis, Investigation, Methodology, Software, Validation, Visualization, Writing – original draft, Writing – review & editing. YL: Funding acquisition, Resources, Writing – review & editing. MF: Funding acquisition, Resources, Writing – review & editing. CL: Conceptualization, Funding acquisition, Resources, Supervision, Writing – review & editing. ZZ: Supervision, Writing – review & editing. HM: Supervision, Writing – review & editing. EX: Supervision, Writing – review & editing. YR: Funding acquisition, Resources, Writing – review & editing.

## References

[B1] Azadbakht M. Ashourloo D. Aghighi H. Radiom S. Alimohammadi A. (2019). Wheat leaf rust detection at canopy scale under different LAI levels using machine learning techniques. Comput. Electron. Agric. 156, 119–128. doi: 10.1016/j.compag.2018.11.016

[B2] Becker F. Choudhury B. J. (1988). Relative sensitivity of normalized difference vegetation Index (NDVI) and microwave polarization difference Index (MPDI) for vegetation and desertification monitoring. Remote Sens. Environ. 24 (2), 297–311. doi: 10.1016/0034-4257(88)90031-4

[B3] Behrens T. Diepenbrock W. (2006). Using Digital Image Analysis to Describe Canopies of Winter Oilseed Rape (Brassica napus L.) during Vegetative Developmental Stages. J. Agron. Crop Sci. 192 (4), 295–302. doi: 10.1111/j.1439-037X.2006.00211.x

[B4] Beriman L. (2001). Random forests. Mach. Learn. 45 (1), 5–32. doi: 10.1023/A:1010933404324

[B5] Broge N. H. Leblanc E. (2001). Comparing prediction power and stability of broadband and hyperspectral vegetation indices for estimation of green leaf area index and canopy chlorophyll density. Remote Sens. Environ. 76 (2), 156–172. doi: 10.1016/S0034-4257(00)00197-8

[B6] Cao Y. Li G. L. Luo Y. K. Pan Q. Zhang S. Y. (2020). Monitoring of sugar beet growth indicators using wide-dynamic-range vegetationindex (WDRVI) derived from UAV multispectral images. Comput. Electronatic. Agric. 171, 105331. doi: 10.1016/j.compag.2020.105331

[B7] Cao Z. Li Y. Huang J. Ye C. Sun B. Shu S.. (2022). Monitoring rice leaf area index based on unmanned aerial vehicle (UAV) digital images. Chin. J. Rice Sci. 36 (3), 308–317. doi: 10.16819/j.1001-7216.2022.210712

[B8] Casa R. Varella H. Buis S. Guérif M. De Solan B. Baret F. (2012). Forcing a wheat crop model with LAI data to access agronomic variables: Evaluation of the impact of model and LAI uncertainties and comparison with an empirical approach. Eur. J. Agron. 37 (1), 1–10. doi: 10.1016/j.eja.2011.09.004

[B9] Chen J. M. Cihlar J. (1996). Retrieving leaf area index of boreal conifer forests using Landsat TM images. Remote Sens. Environ. 55 (2), 153–162. doi: 10.1016/0034-4257(95)00195-6

[B10] Chen T. Guestrin C. (2016). XGBoost: A scalable tree boosting system. ACM.doi: 10.1145/2939672.2939785

[B11] Cui Y. Wei Z. Wang J. Xue Q. (2021). Development status and path of application of chemical fertilizers and pesticides under the background of reduced. Northern Horticulture 000 (009), 164–173.

[B12] Dai M. Yao Z. Liu T. Sun C. (2022). Wheat biomass estimation in different growth stages based on color and texture features of UAV images. Smart Agric. 4, 71–83. doi: 10.12133/j.smartag.SA202202004

[B13] Dash J. Curran P. J. (2004). MTCI: The meris terrestrial chlorophyll index. Int. J. Remote Sens. 25 (549), 151–161. doi: 10.1109/IGARSS.2004.1369009

[B14] Daughtry C. S. T. Walthall C. L. Kim M. S. Colstoun E. B. D. Iii M. M. (2000). Estimating corn leaf chlorophyll concentration from leaf and canopy reflectance. Remote Sens. Environ. 74 (2), 229–239. doi: 10.1016/S0034-4257(00)00113-9

[B15] Dente L. Satalino G. Mattia F. Rinaldi M. (2008). Assimilation of leaf area index derived from ASAR and MERIS data into CERES-Wheat model to map wheat yield. Remote Sens. Environ. 112 (4), 1395–1407. doi: 10.1016/j.rse.2007.05.023

[B16] Dhaliwal S. S. Nahid A. A. Abbas R. (2018). Effective intrusion detection system using XGBoost. Information 9 (7), 149. doi: 10.3390/info9070149

[B17] Du Y. Jiang X. Zhang Y. Huang C. Liu Z. Liu L. (2016). Retrieving leaf area index using PROSAIL radiative transfer model based on Landsat 8 image. Arid Land Geogr. 39, 1096–1103. doi: 10.13826/j.cnki.cn65-1103/x.2016.05.021

[B18] Fu B. He X. Yan H. Liang Y. Deng T. He H.. (2022b). Comparison of RFE-DL and stacking ensemble learning algorithms for classifying mangrove species on UAV multispectral images. Int. J. Appl. Earth Observation Geoinformation 112, 102890. doi: 10.1016/j.jag.2022.102890

[B19] Fu Z. Jiang J. Gao Y. Krienke B. Liu X. (2020). Wheat growth monitoring and yield estimation based on multi-rotor unmanned aerial vehicle. Remote Sens. 12 (3), 508. doi: 10.3390/rs12030508

[B20] Fu B. Sun J. Wang Y. Yang W. He H. Liu L.. (2022a). Evaluation of LAI estimation of mangrove communities using DLR and ELR algorithms with UAV, hyperspectral, and SAR images. Front. Mar. Sci. 9. doi: 10.3389/fmars.2022.944454

[B21] Galvao R. K. H. Araujo M. C. U. Jose G. E. Pontes M. J. C. Silva E. C. Saldanha T. C. B. (2005). A method for calibration and validation subset partitioning. Talanta: Int. J. Pure Appl. Analytical Chem. 67 (4), 736–740. doi: 10.1016/j.talanta.2005.03.025 18970233

[B22] Gao M. Zhang J. Pan Y. Duan Y. Zhang D. (2020). Retrieval of winter wheat leaf area index based on vegetation index and crop height. Chin. J. Agric. Resour. Regional Plann. 41 (8), 49–57.

[B23] Gilabert M. A. González-Piqueras J. Garca-Haro F. J. Meliá J. (2002). A generalized soil-adjusted vegetation index. Remote Sens. Environ. 82 (2), 303–310. doi: 10.1016/S0034-4257(02)00048-2

[B24] Gitelson A. A. Kaufman Y. J. Merzlyak M. N. (1996). Use of a green channel in remote sensing of global vegetation from eos-modis. Remote Sens. Environ. 58 (3), 289–298. doi: 10.1016/S0034-4257(96)00072-7

[B25] Gitelson A. A. Keydan G. P. Merzlyak M. N. (2006). Three-band model for noninvasive estimation of chlorophyll, carotenoids, and anthocyanin contents in higher plant leaves. Geophysical Res. Lett. 33 (11), 431–433. doi: 10.1029/2006GL026457

[B26] Haboudane D. (2004). Hyperspectral vegetation indices and novel algorithms for predicting green LAI of crop canopies: Modeling and validation in the context of precision agriculture. Remote Sens. Environ. 90 (3), 337–352. doi: 10.1016/j.rse.2003.12.013

[B27] Han Y. J. (2011). Influence of global warming on agriculture and its cause analysis, countermeasures. J. Anhui Agric. Sci. 39, 9884–9885+10006. doi: 10.13989/j.cnki.0517-6611.2011.16.023

[B28] Handique B. K. Khan A. Q. Goswami C. Prashnani M. Gupta C. Raju P. L. N. (2017). Crop discrimination using multispectral sensor onboard unmanned aerial vehicle. Proc. Natl. Acad. Sciences India Section A: Phys. Sci. 87, 713–719. doi: 10.1007/s40010-017-0443-9

[B29] Hang Y. Su H. Yu Z. Liu H. Guan H. Kong F. (2021). Estimation of rice leaf area index combining UAV spectrum, texture features and vegetation coverage. Trans. Chin. Soc. Agric. Eng. 37 (9), 64–71. doi: 10.11975/j.issn.1002-6819.2021.09.008

[B30] Hasan U. Sawut M. Chen S. (2019). Estimating the leaf area index of winter wheat based on unmanned aerial vehicle RGB-image parameters. Sustainability 11 (23), 6829. doi: 10.3390/su11236829

[B31] Hassan M. A. Yang M. Rasheed A. Yang G. Reynolds M. Xia X.. (2019). A rapid monitoring of NDVI across the wheat growth cycle for grain yield prediction using a multi-spectral UAV platform. Plant Science. 282, 95–103. doi: 10.1016/j.plantsci.2018.10.022 31003615

[B32] Hochreiter S. Schmidhuber J. (1997). Long short-term memory. Neural Computing 9, 1735–1780. doi: 10.1162/neco.1997.9.8.1735 9377276

[B33] Hu G. Li S. Yang R. (2018). Comparison of three regression models for remote sensing estimation of leaf area index. Sci. Surveying Mapp. 43(10) 46-50, 66. doi: 10.16251/j.cnki.1009-2307.2018.10.007

[B34] Huete A. R. Jackson R. D. Post D. F. (1985). Spectral response of a plant canopy with different soil backgrounds. Remote Sens. Environ. 17 (1), 37–53. doi: 10.1016/0034-4257(85)901117

[B35] Jiang Z. Huete A. R. Didan K. Miura T. (2008). Development of a two-band enhanced vegetation index without a blue band. Remote Sens. Environ. 112 (10), 3833–3845. doi: 10.1016/j.rse.2008.06.006

[B36] Khaki S. Wang L. Archontoulis S. V. (2019). A CNN-RNN framework for crop yield prediction. Front. Plant Sci. 10. doi: 10.3389/fpls.2019.01750 PMC6993602 32038699

[B37] Kim M. S. Daughtry C. S. T. Chappelle E. W. Mcmurtrey J. E. Walthall C. L. (1994). “The use of high spectral resolution bands for estimating absorbed photosynthetically active radiation (A par),” in CNES, Proceedings of 6th International Symposium on Physical Measurements and Signatures in Remote Sensing.

[B38] Koirala A. Walsh K. B. Wang Z. McCarthy C. (2019). Deep learning – Method overview and review of use for fruit detection and yield estimation. Comput. Electron. Agric. 162, 1233. doi: 10.1016/j.compag.2019.04.017

[B39] Kupidura P. (2019). The comparison of different methods of texture analysis for their efficacy for land use classification in satellite imagery. Remote Sens. 11 (10). doi: 10.3390/rs11101233

[B40] Lee S. H. Chan C. S. Wilkin P. Remagnino P. (2015). “Deep-plant: Plant identification with convolutional neural networks,” in Proceedings - International Conference on Image Processing. doi: 10.1109/ICIP.2015.7350839

[B41] Li J. Jiang H. Luo W. Ma X. Zhang Y. (2023). Potato LAI estimation by fusing UAV multi-spectral and texture features. J. South China Agric. Univ. 44 (1), 93–101. doi: 10.7671/j.issn.1001-411X.202201002

[B42] Li Y. Liu H. Ma J. Zhang L. (2021). Estimation of leaf area index for winter wheat at early stages based on convolutional neural networks. Comput. Electron. Agric. 190. doi: 10.1016/j.compag.2021.106480

[B43] Li S. Yuan F. Ata-Ui-Karim S. T. Zheng H. Cao Q. (2019). Combining color indices and textures of UAV-based digital imagery for rice LAI estimation. Remote Sens. 11 (15), 21. doi: 10.3390/rs11151763

[B44] Liang L. Di L. Zhang L. Deng M. Qin Z. Zhao S.. (2015). Estimation of crop LAI using hyperspectral vegetation indices and a hybrid inversion method. Remote Sens. Environ. 165, 123–134. doi: 10.1016/j.rse.2015.04.032

[B45] Liu Y. Feng H. Yue J. Jin X. Li Z. Yang G. (2022). Estimation of potato above-ground biomass based on unmanned aerial vehicle red-green-blue images with different texture features and crop height. Front. Plant Sci. 13. doi: 10.3389/fpls.2022.938216 PMC9452666 36092445

[B46] Liu J. Pattey E. Jégo G. (2012). Assessment of vegetation indices for regional crop green LAI estimation from Landsat images over multiple growing seasons. Remote Sens. Environ. 123, 347–358. doi: 10.1016/j.rse.2012.04.002

[B47] Liu C. Yang G. Li Z. Tang F. Zhang L. (2018). Biomass estimation in winter wheat by UAV spectral information and texture information fusion. Scientia Agricultura Sin. 51, 3060–3073. doi: 10.3864/j.issn.0578-1752.2018.16.003

[B48] Lu Z. Deng L. Lu H. (2022). An improved LAI estimation method incorporating with growth characteristics of field-grown wheat. Remote Sens. 14, 4013. doi: 10.3390/rs14164013

[B49] Luo S. Chen J. Wang C. Gonsamo A. Xi X. Lin Y.. (2018). Comparative performances of airborne LiDAR height and intensity data for leaf area index estimation. IEEE J. selected topics Appl. Earth Observations Remote Sens. 11 (1), 300–310. doi: 10.1109/JSTARS.2017.2765890

[B50] Nevavuori P. Narra N. Lipping T. (2019). Crop yield prediction with deep convolutional neural networks. Comput. Electronic Agric. 163, 104859. doi: 10.1016/j.compag.2019

[B51] Nie S. Wang C. Dong P. Xi X. (2016). Estimating leaf area index of maize using airborne full-waveform lidar data. IEEE J. Selected Topics Appl. Earth Observations Remote Sens. 7 (1-3), 111–120. doi: 10.1080/2150704X.2015.1111536

[B52] Niu Q. Feng H. Yang G. Li C. Yang H. Xu B. (2018). Monitoring plant height and leaf area index of maize breeding material based on UAV digital images. Trans. Chin. Soc. Agric. Eng. 34 (5), 73–82. doi: 10.11975/j.issn.1002-6819.2018.05.010

[B53] Panda S. S. Ames D. P. Panigrahi S. (2010). Application of vegetation indices for agricultural crop yield prediction using neural network techniques. Remote Sens. 2, 673–696. doi: 10.3390/rs2030673

[B54] Pearson R. L. Miller L. D. (1972). Remote mapping of standing crop biomass for estimation of productivity of the shortgrass prairie. Remote Sens. Environment VIII 8. doi: 10.1177/002076409904500102

[B55] Pinter P. J. Hatfield J. L. Schepers J. S. Barnes E. M. Moran M. S. Daughtry C. S. T. (2003). Remote sensing for crop management. Photogrammetric Eng. Remote Sens. 69 (6), 647–664. doi: 10.14358/PERS.69.6.647

[B56] Potgieter A. B. Barbara G. J. Chapman S. C. Kenneth L. Suárez Cadavid Luz A. (2017). Multi-spectral imaging from an unmanned aerial vehicle enables the assessment of seasonal leaf area dynamics of sorghum breeding lines. Front. Plant Sci. 8. doi: 10.3389/fpls.2017.01532 PMC5599772 28951735

[B57] Rondeaux G. Steven M. Baret F. (1996). Optimization of soil-adjusted vegetation indices. Remote Sens. Environ. 55 (2), 95–107. doi: 10.1016/0034-4257(95)00186-7

[B58] Rouse J. W. Haas R. H. Schell J. A. Deering D. W. (1974). Monitoring vegetation systems in the Great Plains with ERTS. NASA Special Publ. 351, 309–317.

[B59] Sun S. Zhao Y. Wang Y. Wang X. Zhang S. (2019). Leaf area index inversion of winter wheat based on multispectral remote sensing of UAV. J. China Agric. Univ. 24 (11), 51–58. doi: 10.11841/j.issn.1007-4333.2019.11.06

[B60] Tao H. Feng H. Xu L. Miao M. Long H. (2020). Estimation of crop growth parameters using UAV-based hyperspectral remote sensing data. Sensors 20 (5), 1296. doi: 10.3390/s20051296 32120958 PMC7085721

[B61] Torres-S´anchez J. Pe˜na J. M. de Castro A. I. L´opez-Granados F. (2014). Multi-temporal mapping of the vegetation fraction in early-season wheat fields using images from UAV. Compututer Electronaic Agric. 103, 104–113. doi: 10.1016/j.compag.2014.02.009

[B62] Wang Q. Putri N. A. Gan Y. Song M. (2022a). Combining both spectral and textural indices for alleviating saturation problem in forest LAI estimation using Sentinel-2 data. Geocarto Int. 37 (25), 10511–10531. doi: 10.1080/10106049.2022.2037730

[B63] Wang J. Si H. Gao Z. Shi L. (2022b). Winter wheat yield prediction using an LSTM model from MODIS LAI products. Agriculture 12 (10), 1707. doi: 10.3390/agriculture12101707

[B64] Yu Z. Ustin S. L. Zhang Z. Liu H. Zhang X. Meng X.. (2020). Estimation of a new canopy structure parameter for rice using smartphone photography. Sensors 20, 4011. doi: 10.3390/s20144011 32707649 PMC7412381

[B65] Yuan Y. Wang X. Yin F. Zhan J. (2013). Examination of the quantitative relationship between vegetation canopy height and LAI. Adv. Meteorology 2013 (3), 1–6. doi: 10.1155/2013/964323

[B66] Zhang Y. Chen J. M. Miller J. R. Noland T. L. (2008). Leaf chlorophyll content retrieval from airborne hyperspectral remote sensing imagery. Remote Sens. Environ. 112 (7), 3234–3247. doi: 10.1016/j.rse.2008.04.005

[B67] Zhang J. Cheng T. Guo W. Xu X. Ma X. (2021a). Leaf area index estimation model for UAV image hyperspectral data based on wavelength variable selection and machine learning methods. Plant Methods 17, 49. doi: 10.1186/s13007-021-00750-5 33941211 PMC8094481

[B68] Zhang J. Qiu X. Wu Y. Zhu Y. Cao Q. Liu X. (2021b). Combining texture, color, and vegetation indices from fixed-wing UAS imagery to estimate wheat growth parameters using multivariate regression methods. Comput. Electron. Agric. 185. doi: 10.1016/j.compag.2021.106138

[B69] Zhang Y. Xia C. Zhang X. Cheng X. Feng G. Wang Y. (2021c). Estimating the maize biomass by crop height and narrowband vegetation indices derived from UAV-based hyperspectral images. Ecol. Indic. 129, 107985. doi: 10.1016/j.ecolind.2021.107985

[B70] Zhang C. Yang G. Li H. Tang F. Liu C. Zhang Y. (2018). Remote sensing inversion of leaf area index of winter wheat based on random forest algorithm. Scientia Agricultura Sin. 51 (5), 855–867. doi: 10.3864/j.issn.0578-1752.2018.05.005

[B71] Zhang X. Zhang K. Sun Y. Zhao Y. Zhuang H. Ban W. (2022a). Combining spectral and texture features of UAS-based multispectral images for maize leaf area index estimation. Remote Sens. 14 (2), 331. doi: 10.3390/rs14020331

[B72] Zhang X. Zhang K. Wu S. Shi H. Sun Y. Zhao Y.. (2022b). An investigation of winter wheat leaf area index fitting model using spectral and canopy height model data from unmanned aerial vehicle imagery. Remote Sens. 14 (20), 5087. doi: 10.3390/rs14205087

[B73] Zheng H. Cheng T. Zhou M. Li D. Yao X. Tian Y. (2018). Improved estimation of rice aboveground biomass combining textural and spectral analysis of UAV imagery. Precis. Agric. 20 (3), 611–629. doi: 10.1007/s11119-018-9600-7

[B74] Zhu Y. Zhao C. Yang H. Yang G. Han L. Li Z.. (2019). Estimation of maize above-ground biomass based on stem-leaf separation strategy integrated with LiDAR and optical remote sensing data. other 7. doi: 10.7717/peerj.7593 PMC6753932 31576235

